# Genome-wide analysis of the CCCH zinc finger family identifies tissue specific and stress responsive candidates in chickpea (*Cicer arietinum* L.)

**DOI:** 10.1371/journal.pone.0180469

**Published:** 2017-07-12

**Authors:** Seema Pradhan, Chandra Kant, Subodh Verma, Sabhyata Bhatia

**Affiliations:** National Institute of Plant Genome Research, Aruna Asaf Ali Marg, New Delhi, India; RIKEN Center for Sustainable Resource Science, JAPAN

## Abstract

The CCCH zinc finger is a group of proteins characterised by a typical motif consisting of three cysteine residues and one histidine residue. These proteins have been reported to play important roles in regulation of plant growth, developmental processes and environmental responses. In the present study, genome wide analysis of the CCCH zinc finger gene family was carried out in the available chickpea genome. Various bioinformatics tools were employed to predict 58 CCCH zinc finger genes in chickpea (designated *CarC3H1-58*), which were analysed for their physio-chemical properties. Phylogenetic analysis classified the proteins into 12 groups in which members of a particular group had similar structural organization. Further, the numbers as well as the types of CCCH motifs present in the CarC3H proteins were compared with those from Arabidopsis and *Medicago truncatula*. Synteny analysis revealed valuable information regarding the evolution of this gene family. Tandem and segmental duplication events were identified and their Ka/Ks values revealed that the *CarC3H* gene family in chickpea had undergone purifying selection. Digital, as well as real time qRT-PCR expression analysis was performed which helped in identification of several *CarC3H* members that expressed preferentially in specific chickpea tissues as well as during abiotic stresses (desiccation, cold, salinity). Moreover, molecular characterization of an important member *CarC3H45* was carried out. This study provides comprehensive genomic information about the important CCCH zinc finger gene family in chickpea. The identified tissue specific and abiotic stress specific CCCH genes could be potential candidates for further characterization to delineate their functional roles in development and stress.

## Introduction

The Zinc finger (Znf) family is one of the largest transcription factor families in eukaryotes [1–4] and is known to regulate genes at the transcriptional or posttranscriptional level [1, 5]. Their binding properties depend on the presence of Zinc finger motifs which are characterised by the presence of cysteines and/or histidines which coordinate a zinc ion to form local peptide structures that facilitate specific biological functions [6]. This superfamily has been divided into nine classes depending upon the number as well as the spacing between the conserved Cys and His residues [7, 8] These classes include C2H2, C8, C6, C3HC4, C2HC, C2HC5, C4, C4HC3, and CCCH. A typical CCCH-type zinc finger protein usually contains one or more zinc finger motifs characterised by three Cys (C) residues followed by one His (H) residue. Based on the different numbers of amino acids present between cysteines and histidines in the CCCH motif, a consensus sequence for these motifs was defined as C-X_4–15_-C-X_4–6_-C-X_3-4_-H (X represents any amino acid) [9]. Most transcription factors regulate gene expression through their DNA or protein binding activity [10]. But most CCCH Znf proteins display RNA binding activity [11–13].

In plants, CCCH genes have been reported to play important roles in cell fate determination and hormone-regulated stress responses. To this effect, a number of members of the Znf-CCCH family have been implicated in various plant developmental and adaptation processes. For example, *AtPEI1* is a well characterized CCCH gene that is indispensable for heart-stage embryo formation in Arabidopsis seeds [14]. Overexpression of another CCCH gene *OsDOS* in rice led to a marked delay in leaf senescence primarily through negative regulation of the jasmonic acid (JA) pathway [3]. Similarly, a cotton CCCH gene, *GhZFP1*, regulates salt tolerance as well as disease resistance by interacting with a dehydration protein and a pathogenesis-related protein respectively [4]. *AtTZF1* has been reported to be involved in sugar signalling [15]. Overexpression of AtTZF1 resulted in compact statured plants, late flowering and higher stress-tolerance. The gene was seen to positively regulate abscisic acid (ABA)/sugar responses and negatively regulate gibberellic acid (GA) responses. The HUA1 protein of Arabidopsis specifically regulates floral morphogenesis by binding to AGAMOUS pre-mRNA [16] while *AtSZF1* and *AtSZF2* negatively regulate the expression of many salt-responsive genes thereby leading to increased salt tolerance in Arabidopsis [17].

The widespread occurrence as well as obvious biological implications has encouraged the study of the CCCH Zn finger family at whole genome level in plants such as Arabidopsis, rice, and maize [9, 18] and more recently in *Medicago* [19], citrus [20] and *Vitis vinifera* [21]. However, despite being the third most important legume in the world, detailed molecular analyses associated with gene families such as the CCCH that regulate important biological processes have not yet been studied in chickpea. Such studies serve to provide a strong foundation for functional characterisation of important genes. Therefore, in this study, CCCH Zn finger genes from the whole genome of chickpea were identified and the members were categorised into groups based on their phylogenetic organisation. Their genomic organisation, as well as the functional domains present in their protein sequences was analysed. Duplication events were also investigated to provide insights into the evolution of this gene family. These genes were subjected to synteny analysis with the genomes of *Medicago*, soybean, *Phaseolus* and Arabidopsis. Their relative expression in different tissues as well as during a number of abiotic stress conditions was investigated. Moreover, one of the members of CCCH family showing the high expression in later stage of seed development was further investigated for expression and regulatory activity.

## Results

### Identification and characterisation of CCCH Znf genes in chickpea

The available desi [22] and kabuli [23] chickpea genomes were used to perform genome wide prediction of CCCH zinc finger transcription factors. The CCCH Znf genes in the chickpea genome were identified using the HMM profile of Zf-CCCH (Zinc finger C-X_8_-C-X_5_-C-X_3_-H type and similar) downloaded from Pfam database (http://pfam.xfam.org/family/PF00642). The presence of CCCH Znf motifs in these predicted proteins was confirmed by performing a domain search on SMART and Pfam. The sequences thus obtained were aligned and manually checked for redundancies. Consequently, a total of 58 non-redundant, full length CCCH Znf transcription factor genes were obtained (named *CarC3H*1-58) and used for all further analysis (S1 Table). The characteristics of these genes, including the number of amino acids present in each gene, their isoelectric point (PI), molecular weight and number of CCCH Znf motifs present in each gene are listed in Table 1. It was observed that the *CarC3H* genes encoded proteins of variable lengths, the longest being CarC3H33 (1915 amino acids) while the smallest encoded 127 amino acids (CarC3H26). The isoelectric points of these proteins ranged from 4.26 (CarC3H8) to 9.67 (CarC3H10).

**Table 1:** Characteristics of CarC3H proteins.

Gene name	Chromosome	Number of amino acids	Molecular weight (Da)	PI	Number of CCCH motifs
CarC3H1	No	488	53319.1	7.18	6
CarC3H2	No	357	40024.5	6.62	4
CarC3H3	No	437	47025	8.28	2
CarC3H4	No	822	90835.7	6.26	3
CarC3H5	No	416	45846	8.11	2
CarC3H6	No	504	55515	5.36	5
CarC3H7	Ca1	712	84586.9	6.47	2
CarC3H8	Ca1	926	105629.3	4.26	1
CarC3H9	Ca1	487	51622.3	8.62	4
CarC3H10	Ca1	179	20066.9	9.67	2
CarC3H11	Ca1	392	44626.7	8.42	2
CarC3H12	Ca1	319	37767.5	9.15	2
CarC3H13	Ca1	1576	171638.4	6.62	1
CarC3H14	Ca2	918	101542.1	6.61	1
CarC3H15	Ca2	396	46807.8	8.84	1
CarC3H16	Ca2	671	73640.2	5.63	1
CarC3H17	Ca2	695	76668.3	6.4	2
CarC3H18	Ca2	422	46257.9	8.27	5
CarC3H19	Ca2	590	67340.7	6.61	1
CarC3H20	Ca3	437	48341.8	8.74	5
CarC3H21	Ca3	464	51289.3	7.28	1
CarC3H22	Ca3	292	31037.1	9.33	3
CarC3H23	Ca3	321	37432.3	9.47	2
CarC3H24	Ca3	328	37098.9	6.29	1
CarC3H25	Ca4	337	37025.8	8.46	5
CarC3H26	Ca4	127	14748.5	8.88	1
CarC3H27	Ca4	426	45213.5	9.06	4
CarC3H28	Ca4	354	39065.4	7.58	2
CarC3H29	Ca4	298	31744	9.39	3
CarC3H30	Ca4	333	38711.7	8.26	2
CarC3H31	Ca4	276	28938	9.2	3
CarC3H32	Ca4	278	32709.2	9.66	2
CarC3H33	Ca4	1915	212135.1	6.69	4
CarC3H34	Ca5	704	76647.4	5.65	1
CarC3H35	Ca5	463	52270.6	5.58	4
CarC3H36	Ca5	726	78504.7	5.77	1
CarC3H37	Ca5	631	69978.2	5.35	1
CarC3H38	Ca5	472	53043.2	5.28	5
CarC3H39	Ca5	352	40104.5	7.91	1
CarC3H40	Ca5	854	94615.4	9.06	3
CarC3H41	Ca6	385	42903.9	6.64	2
CarC3H42	Ca6	816	90714.1	8.68	3
CarC3H43	Ca6	673	75233.1	5.72	3
CarC3H44	Ca6	214	23556.2	9.39	1
CarC3H45	Ca6	263	30093.9	9.16	2
CarC3H46	Ca6	375	41150.3	6.61	1
CarC3H47	Ca6	789	86487.7	5.76	1
CarC3H48	Ca6	507	58225.6	7.04	1
CarC3H49	Ca7	585	65567	6.79	2
CarC3H50	Ca7	454	47406.9	8.38	4
CarC3H51	Ca7	520	59167	6.15	3
CarC3H52	Ca7	421	46951.5	9.15	3
CarC3H53	Ca7	381	40749.5	6.83	3
CarC3H54	Ca7	473	53789.4	9.05	1
CarC3H55	Ca7	428	47064.7	8.51	2
CarC3H56	Ca7	689	74984.3	5.81	1
CarC3H57	Ca8	657	72345.9	6.07	1
CarC3H58	Ca8	365	40701.5	7.52	3

**Fig 1:**
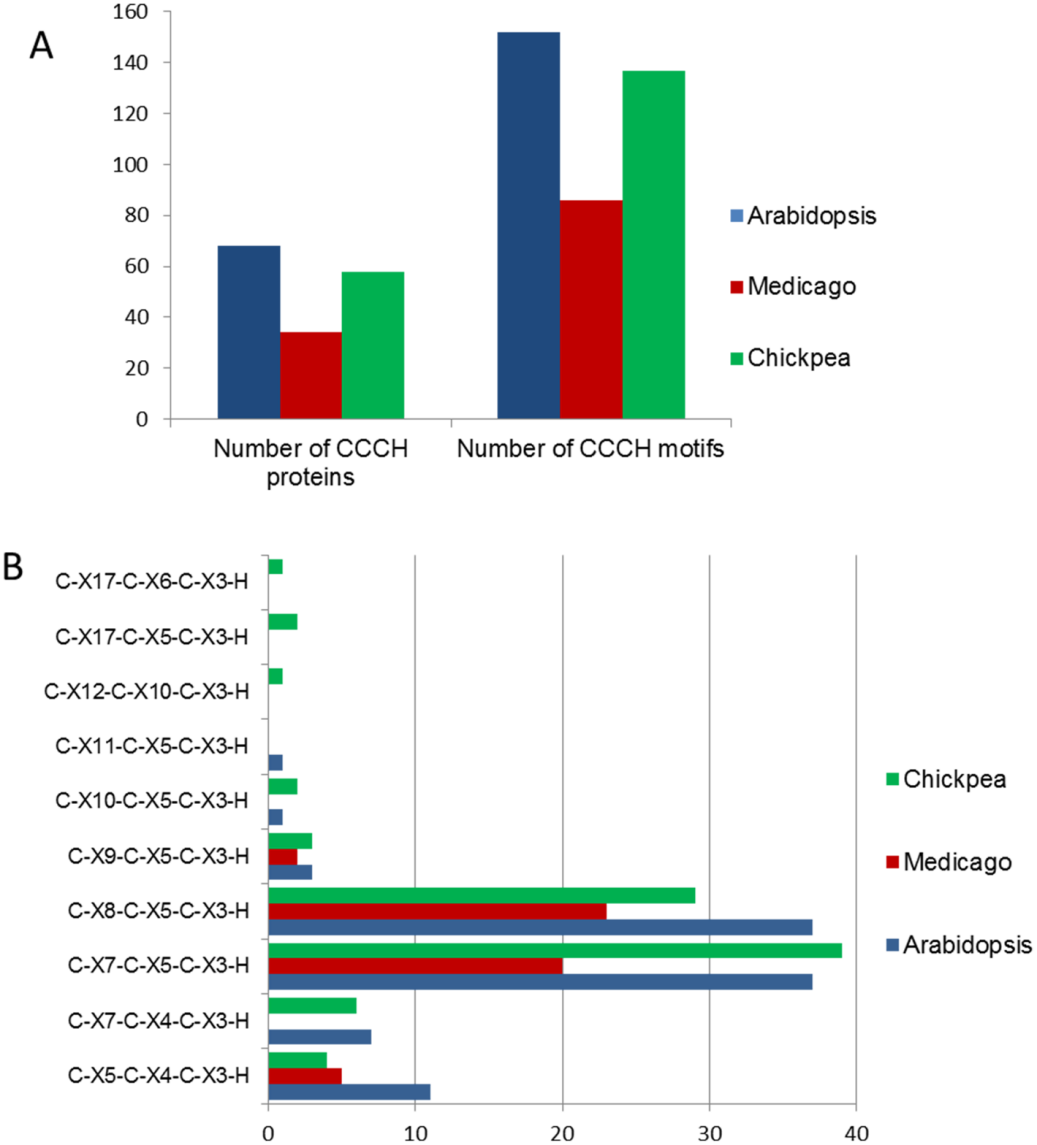
Comparison of CCCH ZnF containing TFs in Arabidopsis, *Medicago* and chickpea. (A) Total number of CCCH proteins and CCCH motifs present in the three plants. (B) Distribution of CCCH motifs into various consensus arrangements in the three plants

SMART and Pfam databases were used to calculate the total number of CCCH Znf motifs in the CarC3H proteins and a total of 138 CCCH Znf motifs were identified in this study. The members of chickpea CCCH Znf gene family were found to have 1 to 6 C3H type Znf domains. Majority of the members had either one (20 members) or two (15 members) C3H domains while 11 members contained three C3H domains and 12 members contained more than three C3H Znf domains (Table 1). Comparison with the CCCH Znf genes reported from Arabidopsis and *M*. *truncatula* showed that the number of CCCH Znf genes in chickpea was greater than those reported in *M*. *truncatula* while lower than Arabidopsis (Fig 1A). The numbers of CCCH motifs in the three plants were seen to vary accordingly, with Arabidopsis containing the highest number of CCCH motifs, followed by chickpea and *M*. *truncatula* (Fig 1A). The MEME program was used to identify all the motifs present in the CarC3H protein sequences. This led to prediction of a total of 10 different motifs including Znf-CCCH (Table 2). Similar to results observed in Arabidopsis and *M*. *truncatula*, the most common types of CCCH motifs observed were C-X_8_-C-X_5_-C-X_3_-H and C-X_7_-C-X_5_-C-X_3_-H type CCCH motifs (Fig 1B). A few members (CarC3H7 and CarC3H16) were also seen to contain a CCCH motif with the constitution C-X_10_-C-X_5_-C-X_3_-H. In addition, some members were found to contain unconventional CCCH motifs. For example, CarC3H20 and CarC3H25 contained a CCCH motif with the constitution C-X_17_-C-X_5_-C-X_3_-H while CarC3H30 contained a motif with C-X_17_-C-X_6_-C-X_3_-H as its consensus (S2 Table).

**Table 2:** Prediction of conserved motifs in CarC3H proteins.

Motif No.	Motif length	No. of sites	Motif sequence	E-value
1	21	50	CS[YF]Y[LM][KR]TGxCKFG[SA][TS]C[KR][FY][HND]HP	2.5e-626
2	21	50	PCRFF[AR][KR]GSCR[RK]GDxC[ER][FY][ALS]HG	1.0e-401
3	41	16	[MP][AYF][SEQ][FY][KR][VT]R[PLR]C[KS][RD][GA][YRT]S[HC]D[WR][TR][EV]C[PF]F[AV]HPGE[NEK][AL]R[RP][RL][DY]P[RS][KT][FYG][HS][YA][SV][CGP]	7.5e-310
4	26	10	[SVW]K[ST][KR][ILP]C[NTK]K[FW][FNEY][ST][TA][EGS]GC[PK][FY]G[DE][KGS]CHF[AL]H[GY]	9.1e-107
5	50	4	[VT][QEL]DVRIP[CY]Q[QE][KR]RMFGFVTF[VA][DFY]P[EK]TV[KR][MIL]I[LF][ADES][KN]GNPH[FY][VI][CH][DG][SA]RVLVKPY[KR]E[KR]	8.7e-077
6	50	5	[YL]I[LI][SAGL][TCY][GP][EKRY][VA][DH]VN[RF][SARV][CS]G[STV][DN][GKM][AS]TALHCA[VA][ASF]G[GC]S[AVE][NAF][SAV][VILP][ED][IVA][VI][KR]LL[LI][DS]A[GS]A[DE][AVI][NSD][CAS][VL]	1.4e-061
7	15	30	LLNS[LE]G[LYF]P[EL]RPG[EQ]P[ED]	2.8e-055
8	21	15	[IVP][VG][GW][DGS][DN][KM][LF][FYL][STA]G[SD]T[DT]GT[ILV][RK]VW[DKN][ACLW]	2.0e-058
9	50	4	[HC]F[EDK][DE]FY[ED]D[LIV][FH][ELT]E[LF][SL]K[FY]G[DEQY][IL][EQV][SNT][LF][NK][VI]C[DK]N[LG][AS][DF]H[ML][VIR]GNVYV[QL][FY][KR][EL][EL][DE][HS]A[AL][NAR]A[LVY]	1.4e-051
10	15	13	EVEEEEEEEE[EV]EEEE	4.0e-049

### Phylogenetic classification and structural organisation of *CarC3H* gene family

**Fig 2:**
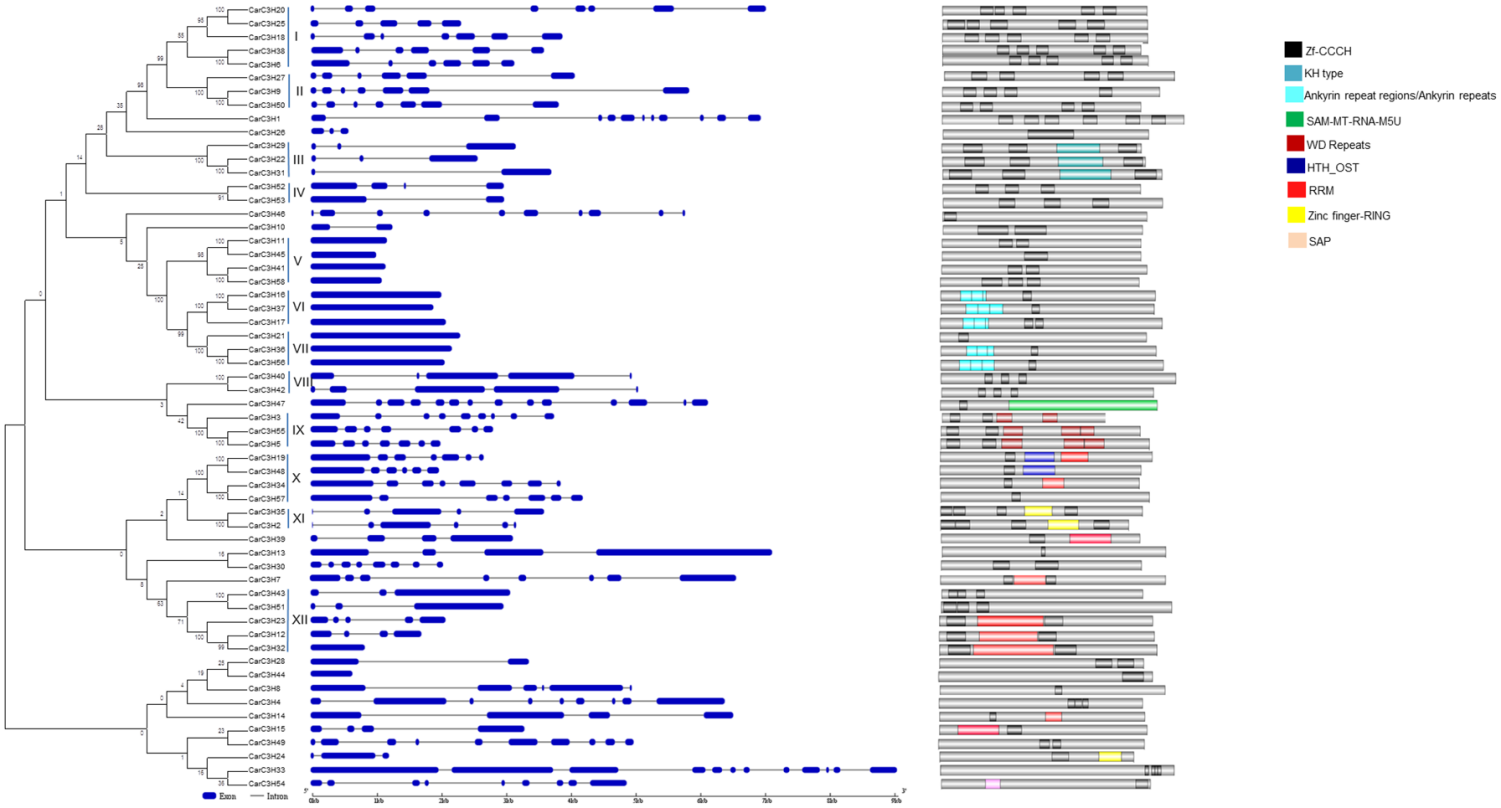
Phylogenetic classification of *CarC3H* members. The CarC3H members were divided into 12 groups based on their phylogenetic positions. The structural similarity of their corresponding exon-intron organisation as well as distribution of functional domains in their amino acid sequences validates this clustering

The amino acid sequences of the *CarC3H* genes were aligned and used to generate the phylogenetic tree (Fig 2) that revealed that the chickpea C3H Znf genes were divided into twelve groups designated I-XII (Fig 2). Most of the clusters were supported by high bootstrap values in the phylogenetic analysis which validated the prediction and alignment of the *CarC3H* genes. To further substantiate the phylogenetic data, the CarC3H genes were aligned to the kabuli chickpea genome [23] and their structural organisation based on the exon-intron arrangement was deduced. It was observed that most of the CCCH genes in a particular group had similar genomic organisation. For example, all the members of group V, VI and VII were intron-less while members of the adjacent group VIII had four introns and five exons each. Members of group IX and XI exhibited similarity in the numbers of introns and exons present in their genes. Despite a number of deviations from this trend, where the numbers of exons and introns amongst members of a group were different, the pattern of genomic organisation was remarkably well conserved.

The phylogenetic classification was further reinforced through prediction of functional domains in the proteins of respective *CarC3H* genes (Fig 2). The CCCH Znf proteins are known to regulate gene expression through a number of methods including post transcriptional modification of target pre-mRNAs [24, 25], transcriptional activation or repression of target genes [1, 5] and interaction with different proteins [4]. This regulation is facilitated by the presence of various functional domains in addition to the CCCH motifs. The functional motifs found in members of CarC3H family included motifs like K homolog domains (KH type) and RRM motifs that are involved in RNA processing and Ankyrin, WD repeats and Zf-RING motifs that are involved in protein-protein interactions. In addition, some members were seen to contain domains like HTH-OST, SAM-MT-RNA-M5U and SAP. Schematic representation of various domains in the CarC3H protein sequences showed that members of a group displayed remarkable similarity in the type of domains present (Fig 2). For example, all members of group III were seen to contain KH type domain while members of group IX and XI were found to possess WD repeats and Zf-RING domains respectively.

**Fig 3:**
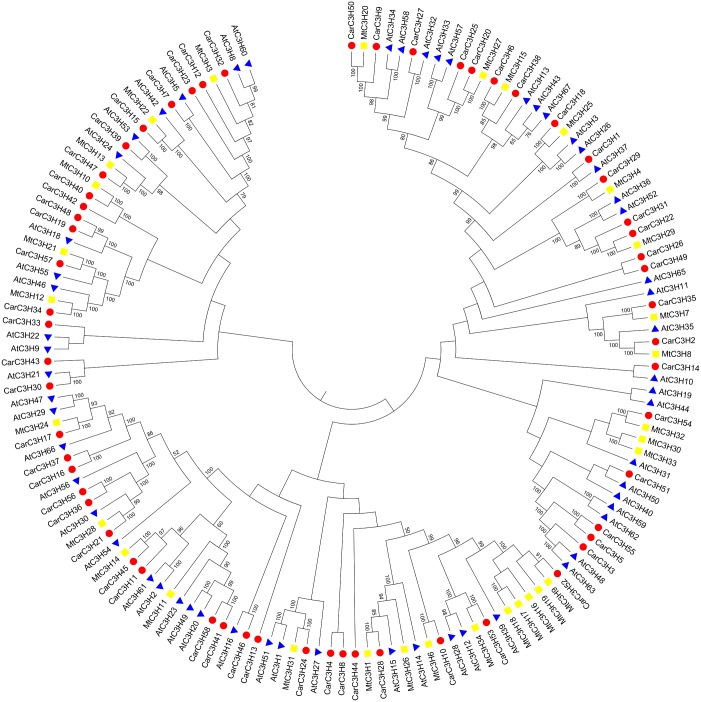
Phylogenetic comparison of CCCH Znf proteins in chickpea, Arabidopsis and *Medicago*.

Phylogenetic analysis of members of CCCH Znf gene families of chickpea along with members from *M*. *truncatula* and Arabidopsis was also carried out (Fig 3). It was observed that most members of chickpea C3H gene family were phylogenetically very close to their homologs from *Medicago* and Arabidopsis. For example, some pairs of CarC3H and AtC3H genes like CarC3H27/AtC3H32, CarC3H1/AtC3H37, CarC3H30/AtC3H21, CarC3H7/AtC3H5 and CarC3H39/AtC3H53 were very closely related. Similarly, a number of CarC3H members showed very close homology with their homologs from *M*. *truncatula*. The amino acid sequences of these gene pairs were analysed and it was observed that they shared similar domains in their protein sequences. For example, SAP domain was present in the amino acid sequences of both CarC3H54 and MtC3H32 whereas both CarC3H22 and MtC3H29 contained a KH type domain thereby clearly validating the phylogenetic arrangement of the CarC3H members in the tree.

### Synteny analysis

**Fig 4:**
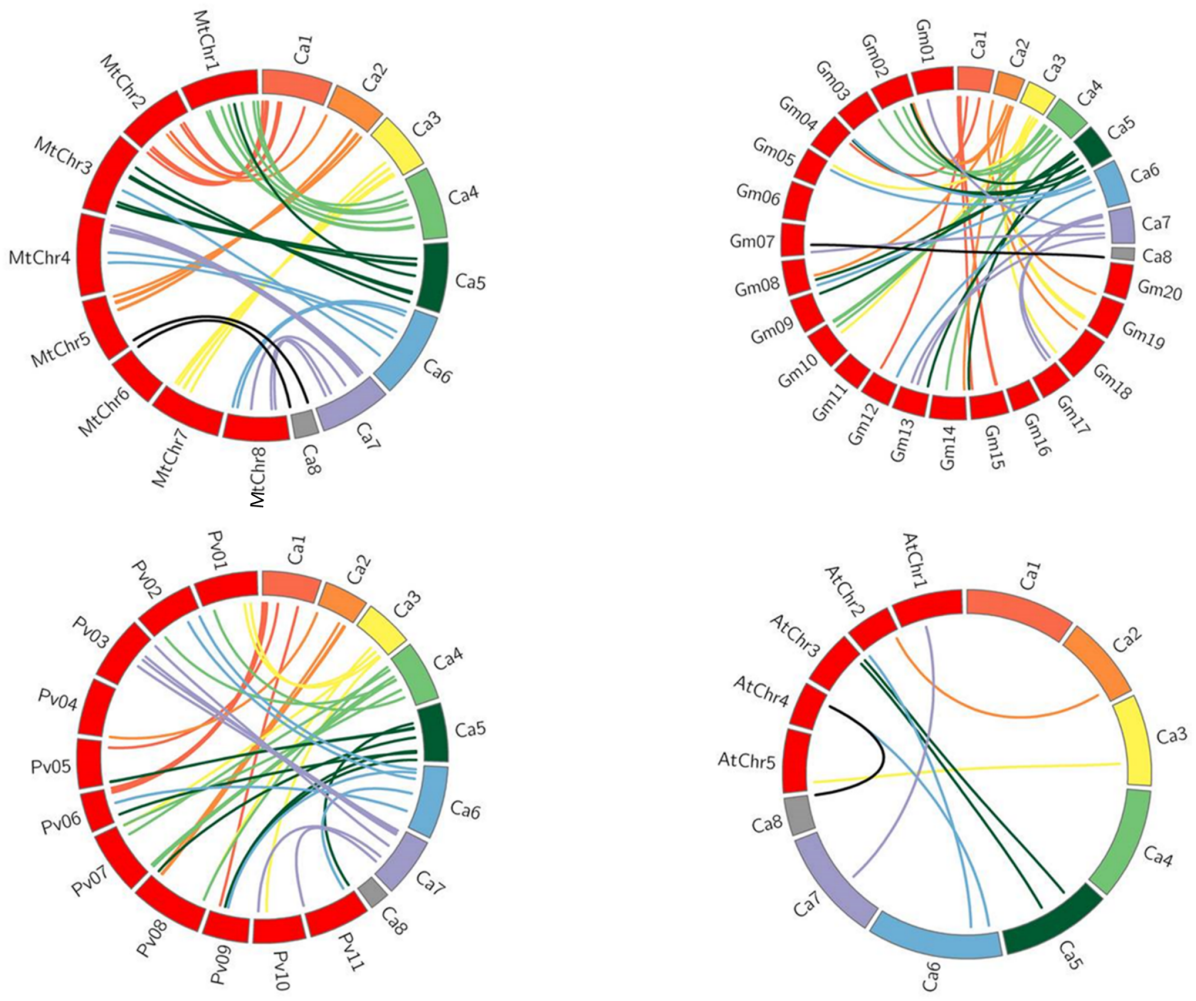
Synteny analysis of *CarC3H* genes with the genomes of *M*.*truncatula*, *G*.*max*, *P*. *vulgaris and A*. *thaliana*.

Synteny may be defined as the conserved order of genes on chromosomes of related species as a result of descent from a common ancestor and comparative mapping is a valuable technique to identify similarities and differences between species [26]. Therefore, the *CarC3H* sequences were mapped onto the genomes of *M*. *truncatula*, *G*. *max*, *P*. *vulgaris* and Arabidopsis. This comparative analysis revealed that 52 members of *CarC3H* gene family found homologs in the *M*. *truncatula* genome, 48 members found homologs in *G*. *max* genome, 43 *CarC3H* genes found homologs in *P*. *vulgaris* genome and only 8 *CarC3H* genes could be mapped onto the *A*. *thaliana* genome (Fig 4). These results reaffirmed the common ancestry shared by chickpea and *M*. *truncatula* and *G*. *max* Similar inferences have been derived based on a number of studies which report that both chickpea and *M*. *truncatula* belong to the Galegoid group of legumes and hence share a closer phylogenetic relationship [27].

### Chromosomal location and duplication of *CarC3H* genes

**Fig 5:**
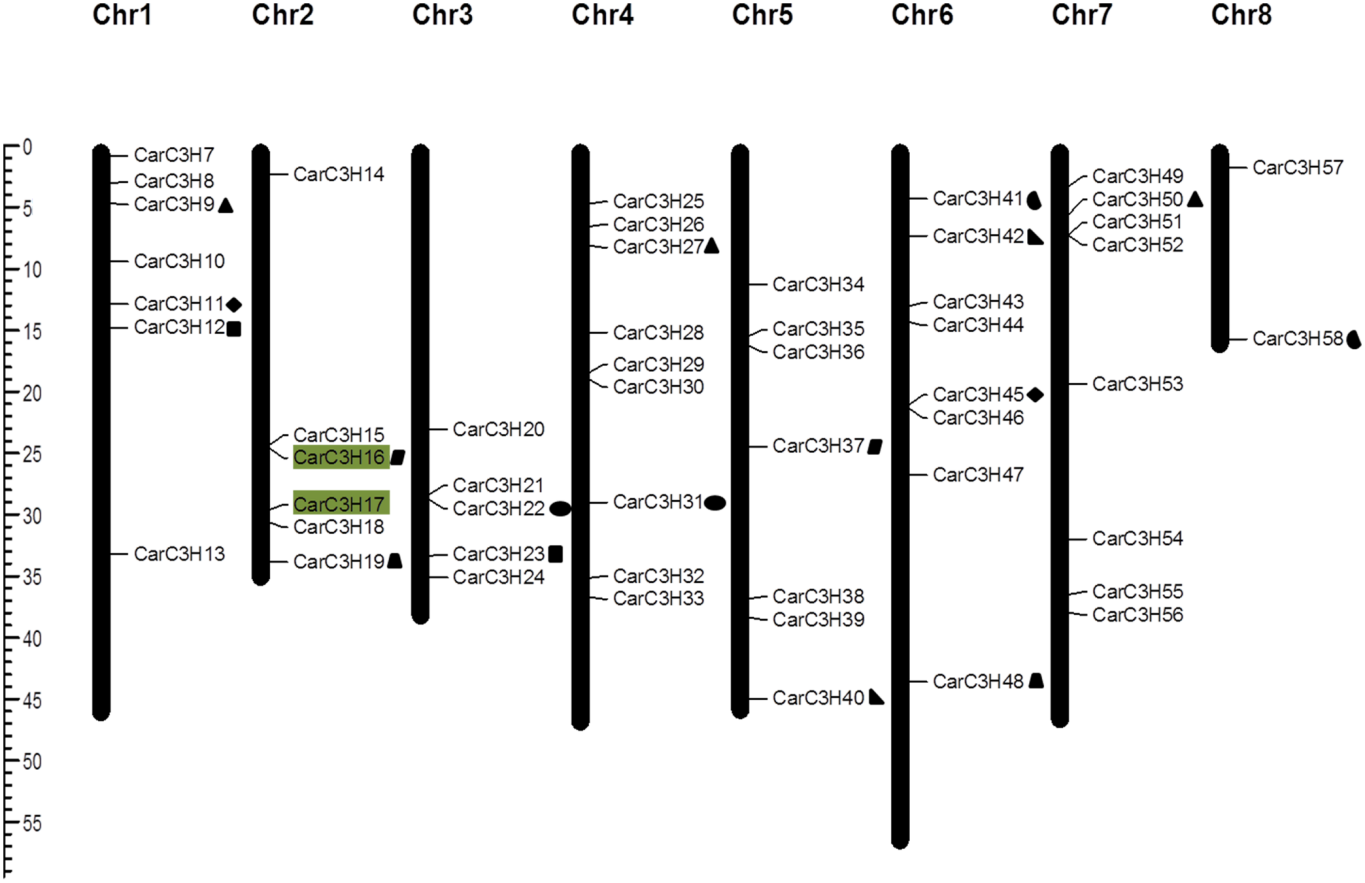
Location of *CarC3H* genes on the chromosomes of kabuli chickpea and duplication events in members of *CarC3H* gene family. Tandemly duplicated genes are highlighted with same colour background and segmental duplications are represented by the type of blocks against duplicated members

The *CarC3H* genes were mapped onto the kabuli chickpea genome [23] in order to assign their chromosomal positions (Fig 5). Fifty-two *CarC3H* genes were located on the 8 chickpea chromosomes whereas 6 members (*CarC3H*1-*CarC3H*6) mapped onto the unanchored scaffolds of the kabuli chickpea genome. However, keeping in mind their size and their similarity to known CCCH genes, these have been included in all analyses. Maximum number (9) of *CarC3H* genes were located on Chromosome 4 while Chromosome 8 contained the least (2). Chromosomes 6 and 7 contained 8 *CarC3H* genes each while Chromosomes 1 and 5 accounted for 7 *CarC3H* genes each. Chromosomes 2 and 3 contained 6 and 5 *CarC3H* genes respectively (Fig 5).

Determination of duplication events revealed 10 pairs of paralogous *CarC3H* genes distributed on various chromosomes (Fig 5). One of these events was found to be tandem gene duplication (Fig 5). On the other hand, nine duplication events involved gene pairs with segmental duplications which occur at more than one site within the genome and typically share a high level of sequence identity [28]. According to Wagner [29] accumulation of advantageous mutations usually leads to divergence of species and hence is known as positive or diversifying selection while removal of deleterious mutation leads to survival of a species and hence is called negative or purifying selection. Diversifying or purifying nature of a selection can be determined by comparison of synonymous (Ks) and non-synonymous (Ka) rates of substitution of bases [30, 31]. Hence, this method was adopted to generate the Ka/Ks values for the 10 pairs of paralogous genes (Table 3). The results indicated that the *CarC3H* genes were under purifying selection since the Ka/Ks values for all gene pairs was less than 1.

**Table 3:** Details of duplicated *CarC3H* genes and their Ka/Ks vaalues.

Gene pair	Type of duplication	Ka	Ks	Ka/Ks
CarC3H9/27	Segmental	0.4417	2.3192	0.1905
CarC3H9/50	Segmental	0.187	0.5518	0.3388
CarC3H11/45	Segmental	0.4965	3.6895	0.1346
CarC3H12/23	Segmental	0.3229	7.9436	0.0406
CarC3H16/17	Tandem	0.3639	3.1524	0.1154
CarC3H16/37	Segmental	0.2785	0.9755	0.2855
CarC3H41/58	Segmental	0.1714	1.2462	0.1375
CarC3H19/48	Segmental	0.5385	2.1185	0.2542
CarC3H40/42	Segmental	0.4197	0.9491	0.4421
CarC3H22/31	Segmental	0.1446	0.742	0.1948

### Tissue specific expression of *CarC3H* genes

**Fig 6:**
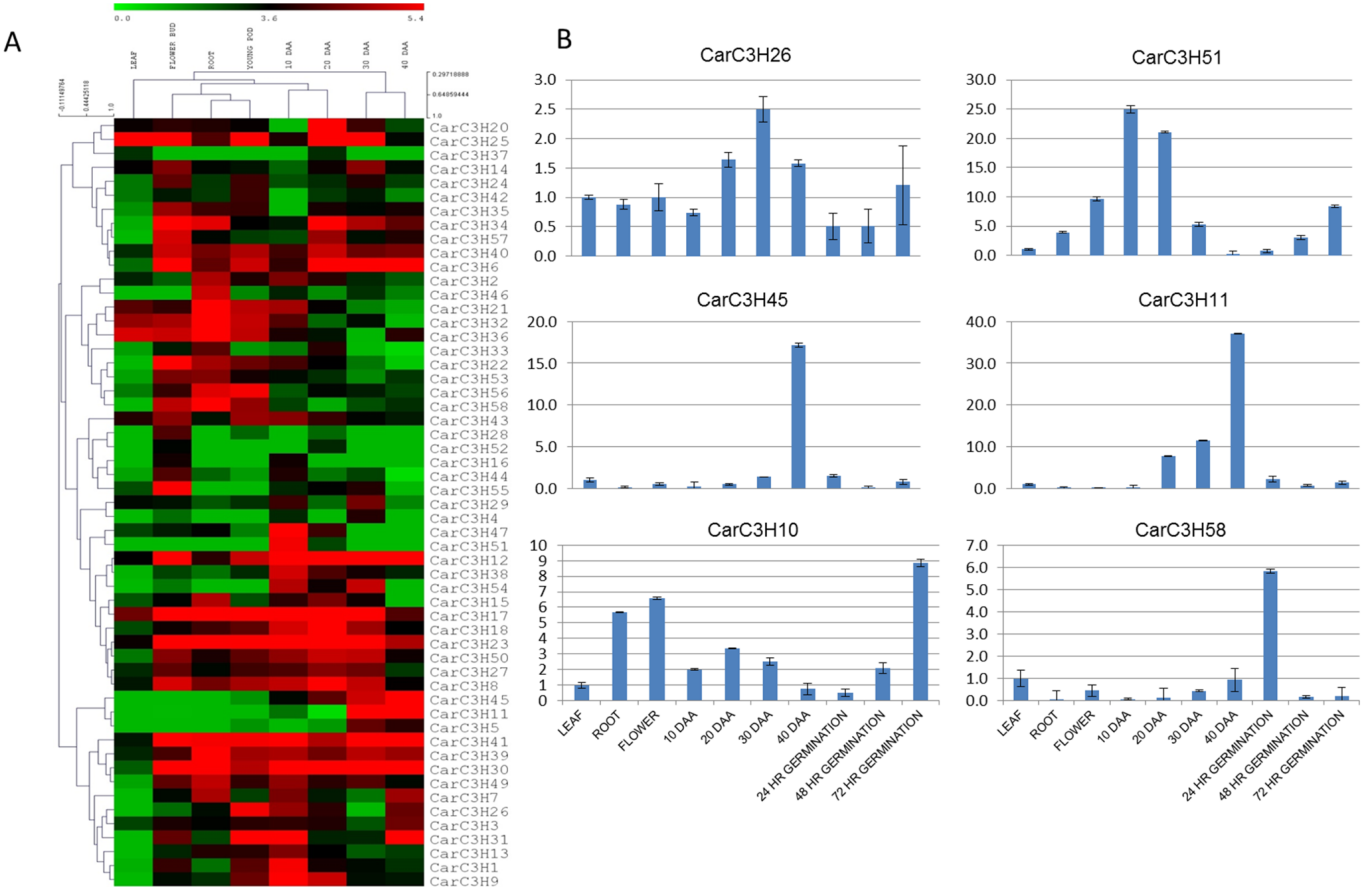
**(A)** Digital expression analysis of *CarC3H* genes in different tissues of chickpea. Scale represents the log_2_ normalised RPKM values. (**B)** qRT PCR based analysis of CarC3H genes having preferential expression in chickpea seed tissue. Values on the Y-axis indicate relative expression values as calculated by 2 –ΔΔCt method

The availability of deep transcriptomes of different chickpea tissues (leaf, root, flower bud, young pod) [32] as well as the transcriptome of chickpea seeds at various stages of development [33], allowed analysis of the tissue specific expression of the *CarC3H* genes. The raw 454 sequence reads from 8 different chickpea tissues (leaf, root, flower bud, young pod, 10DAA seed, 20DAA seed, 30DAA seed and 40DAA seed) were mapped onto the *CarC3H* genes and RPKM values were calculated based on the number of raw reads mapped onto each tissue. These RPKM values obtained were log_2_ normalized and used to generate a heat map depicting the digital expression pattern of the *CarC3H* genes across different chickpea tissues. The samples were clustered according to their corresponding expression patterns using hierarchical clustering (Fig 6A). The analysis showed that *CarC3H28*, *52*, *16*, *44* and *55* were highly expressed in the flower bud. *CarC3H46* was seen to have especially high expression in root tissue, while *CarC3H21*, *32*, *36*, *33* and *58* were observed to have comparatively higher expression in root. Also a number of *CarC3H* genes were found to be expressed highly in chickpea seed tissue. For example, *CarC3H47* and *CarC3H51* were expressed at higher levels in early stages of seed development (10 and 20 DAA) while *CarC3H11* and *CarC3H45* had greater expression levels in later stages (30 and 40 DAA). In addition, the promoter sequences for some genes contained motifs which correlate with their tissue specific expression. For example, the motif “CARGCW8GAT”, which is a binding site for AGL15, was found in promoter sequences of *CarC3H16*, *44* and *55* and responsible for their expression in flower bud and seed. Genes with higher expression in seed tissue contained promoter motifs like “RYREPEAT”, “SEF1” and “SEF4”, which are known to impart seed specific expression of genes (S3 Table). On the other hand, a large number of genes contained promoter elements that did not correlate with their tissue specific expressions. Quantitative real time PCR was used to further analyse and validate the expression of *CarC3H* genes in various tissues of chickpea, including germinating seedling at 24hr, 48hr and 72hr of germination. According to this analysis, *CarC3H26*, *CarC3H11*, *CarC3H45* and *CarC3H51* had higher expression in seed tissue while *CarC3H10* and *CarC3H58* had higher expression in germinating chickpea seedlings (Fig 6B).

### Expression of CarC3H genes under abiotic stress conditions

**Fig 7:**
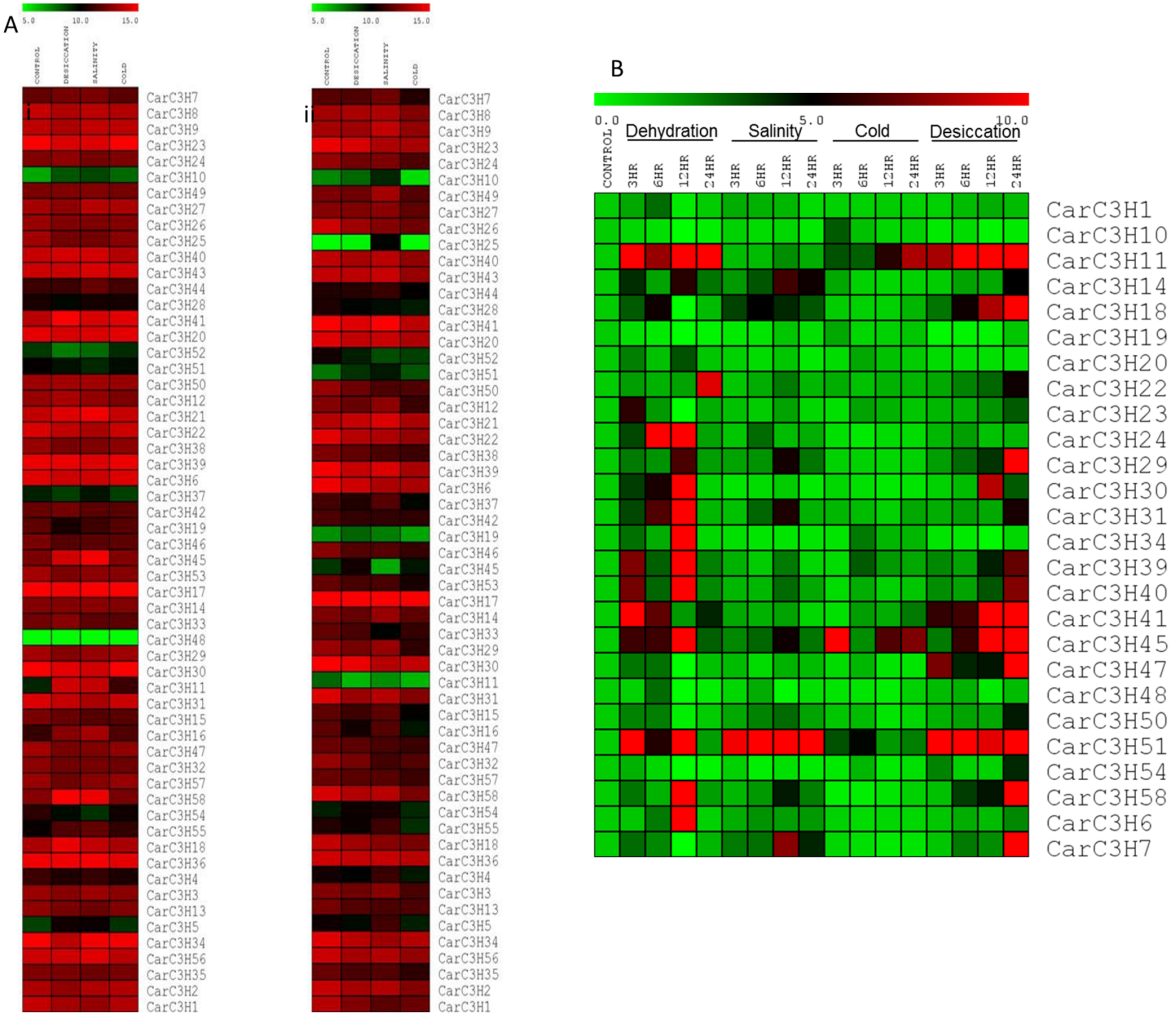
**(A)** Digital expression analysis of *CarC3H* genes in control and stressed tissues. Scale represents the log_2_ normalised RPKM values. (**B)** qRT-PCR based expression analysis of *CarC3H* genes in control and stressed chickpea seedlings (3 weeks old) at different time points. Scale represents the relative expression values as calculated by 2 –ΔΔCt method.

To study the effect of various abiotic stresses on the expression of *CarC3H* genes, the raw reads generated by sequencing of chickpea shoot and root tissue subjected to different abiotic stresses (desiccation, salinity and cold) were downloaded from NCBI SRA database (SRX402843, SRX402845, SRX402844, SRX402846, SRX402842, SRX402841, SRX402840, SRX402839) and mapped onto *CarC3H* gene sequences. The RPKM values were calculated based on the number of raw reads that mapped and log_2_ normalised to generate the heat map (Fig 7A). It was observed that most of the *CarC3H* genes had high expression in both control and stressed tissues. However, *CarC3H11*, *16*, *45*, *58*, *55* and *5* were found to have higher expression in chickpea shoots subjected to desiccation and salinity stress (Fig 7A). In case of roots, *CarC3H25*, *55*, *4* and *5* were found to have higher expression under salinity stress. *CarC3H19*, *45* and *11* had high expression in shoot during desiccation, salinity and cold stresses while expressing at very low levels in root. It was also observed that cold stress had no significant effect on the expression of the *CarC3H* genes.

To further analyse and validate their expression under abiotic stress, some of the *CarC3H* genes were chosen for quantitative real time PCR. Three-week old chickpea seedlings (cv. ICCV2) were subjected to dehydration, salinity, cold and desiccation stresses and samples were collected at various time points. Quantitative real time PCR analysis showed that dehydration and desiccation stresses had a pronounced effect on expression of *CarC3H* genes as compared to salinity and cold (Fig 7B). These results indicated the putative involvement of the members of groups III and V of *CarC3H*gene family which included *CarC3H29*, *22*, *31* (group III) and *CarC3H11*, *41*, *45 and 58* (group V). The genes in these groups showed higher expression under dehydration and desiccation stresses (Fig 7B. The promoters of these genes were found to contain motifs like “ABRELATERD1” and “ACGTATERD1”, which may drive their expression in response to dehydration (S3 Table).

### Molecular characterization of the CarC3H45 gene

#### Amplification and copy number determination for CarC3H45 gene

**Fig 8:**
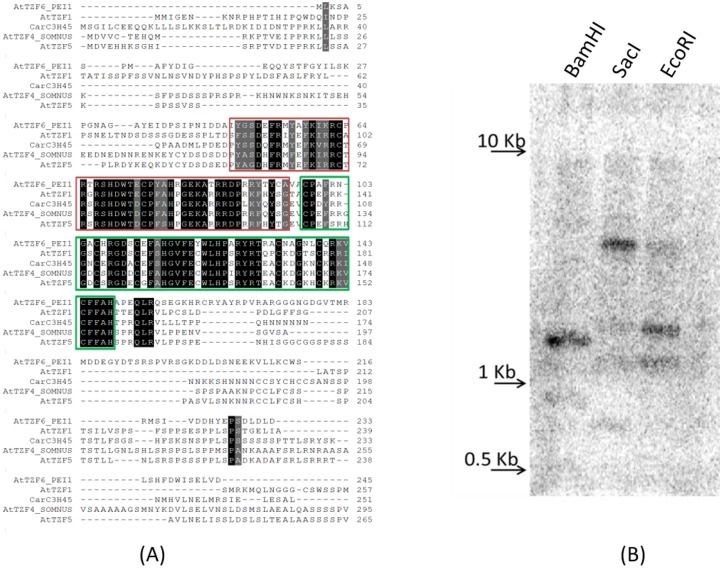
**(A)** Alignment of CarC3H45 protein sequence with known tandem CCCH zinc finger proteins from *Arabidopsis*. Sequence within green box indicates the CCCH motif and red box indicates conserved arginine rich region. **(B)** Southern blotting analysis showed that the *CarC3H45* was present as a single copy in chickpea.

Amongst the CarC3H members differentially expressed across various tissues and stress conditions, an interesting member *CarC3H45* (a group V member), showed significant expression in seed tissue as well as during dehydration and desiccation stress. It was therefore selected for further characterisation. Sequencing of the coding region revealed that the ORF for *CarC3H45* gene was 996bp long that coded for a protein of 331 amino acids. The ORF was mapped onto the whole genome sequence of chickpea to determine its genomic organisation. The*CarC3H45* gene was found to be intronless. Moreover, CarC3H45 was found to consist of two CCCH zinc finger motifs (Table 1), which was similar to the reported tandem zinc finger proteins in *Arabidopsis* (AtTZF1, AtTZF4, AtTZF5 and AtTZF6), which have been studied for their role in seed development [34, 35, 36]. Further the tandem CCCH Znf motif was conserved across the four Arabidopsis as well as the CarC3H45 with the consensus being C-x7–8-C-x5-C-x3-H- x16-C-x5-C-x4-C-x3-H (Fig 8A). In addition, it was observed that a conserved arginine rich region was also found to be present upstream of the CCCH Znf motif.

In order to determine the copy number of *CarC3H45* gene in *C*. *arietinum* cv. ICCV2 southern blotting was carried out. A part of the *CarC3H45* gene excluding the conserved tandem CCCH coding region was used as a probe for hybridisation. A prominent single band was observed after hybridisation in lanes where the genomic DNA had been digested with *Bam*HI and *Sac*I (both of which did not have restriction site in the gene). Whereas, genomic DNA digested with *Eco*RI (that has a restriction site within the gene) produced two bands (Fig 8B). This clearly indicated that the *CarC3H45* was present as a single copy in the chickpea genome.

#### Quantitative expression analysis

**Fig 9:**
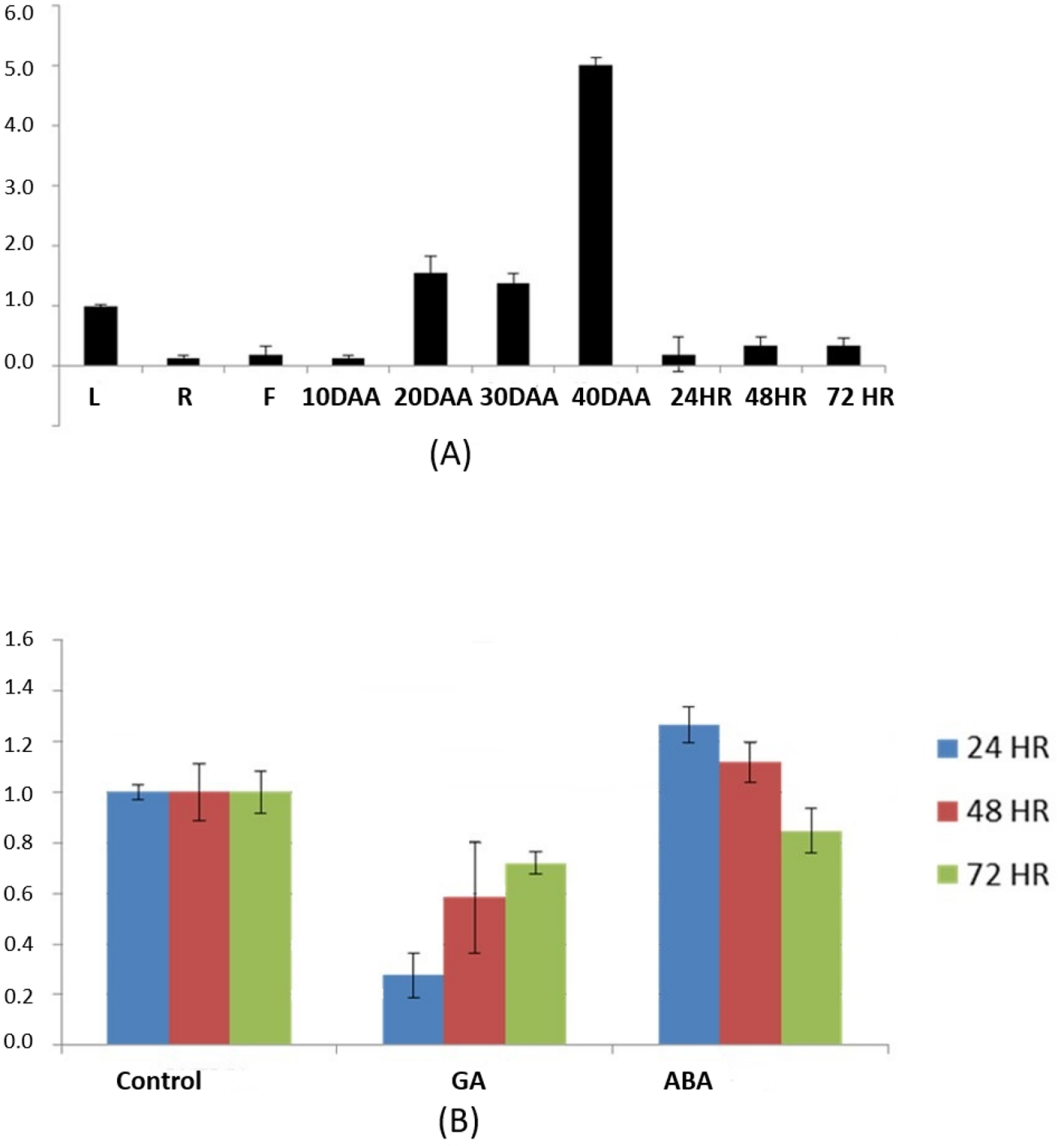
Quantitative expression of *CarC3H45* in (A) different tissues of chickpea, L = leaf, R = root, F = flower, DAA = Days after anthesis, 24 Hr = 24 hours old seedling, 48 Hr = 48 hours old seedling and 72 Hr = 72 hours old seedling and (B) in germinating seeds treated with GA and ABA at various time points.

Expression patterns of CarC3H45 were analysed across tissues, various stages of developing seeds and under ABA and GA treatment. The qRT-PCR analysis showed that *CarC3H45* was expressed significantly in the mature seed (40 DAA) (Fig 9A). It was also observed that although the gene had high expression in 40 DAA seed tissue, it had much lower expression in germinating seedlings of chickpea suggesting its involvement in seed maturation and dormancy. In plants abscisic acid (ABA) and gibberellic acid (GA) are known to regulate maturation and dormancy, therefore mature seeds of chickpea were subjected to ABA and GA treatment and the level of expression of *CarC3H45* was determined using qRT PCR. The results showed that the expression of *CarC3H45* increased upon application of ABA and decreased under the influence of GA (Fig 9B).

#### Promoter analysis and transactivation activity

Motifs present in the promoter regions of genes play crucial roles in determining their regulation. About 1500bp region upstream of the *CarC3H45* ORF was amplified using specific primers (S4 Table), cloned and sequenced. Analysis showed the presence of a number of important motifs in the promoter region of *CarC3H45* as had been predicted *In-silico* (S3 Table). One of the motifs was the RY Repeat element which is known to bind of B3 domain containing proteins transcription factors implicated in seed development [37, 38]. Previous studies have also reported that ABI3, which is a B3 domain containing protein and one of the master regulators of seed development can regulate CCCh-Znf protein SOMNUS [39]. Therefore, yeast one hybrid analysis was performed to investigate the interactions between *CarC3H45* and ABI3. Results showed that the elevated aureobasidin levels had little or no effect on the growth of colonies containing pGADT7-CarABI3/pAbAi-*C3H45Prom* while the rest of the samples showed reduced or no growth at high concentrations of the antibiotic. This suggested the putative role of CarABI3 in regulation of *CarC3H45*.

**Fig 10:**
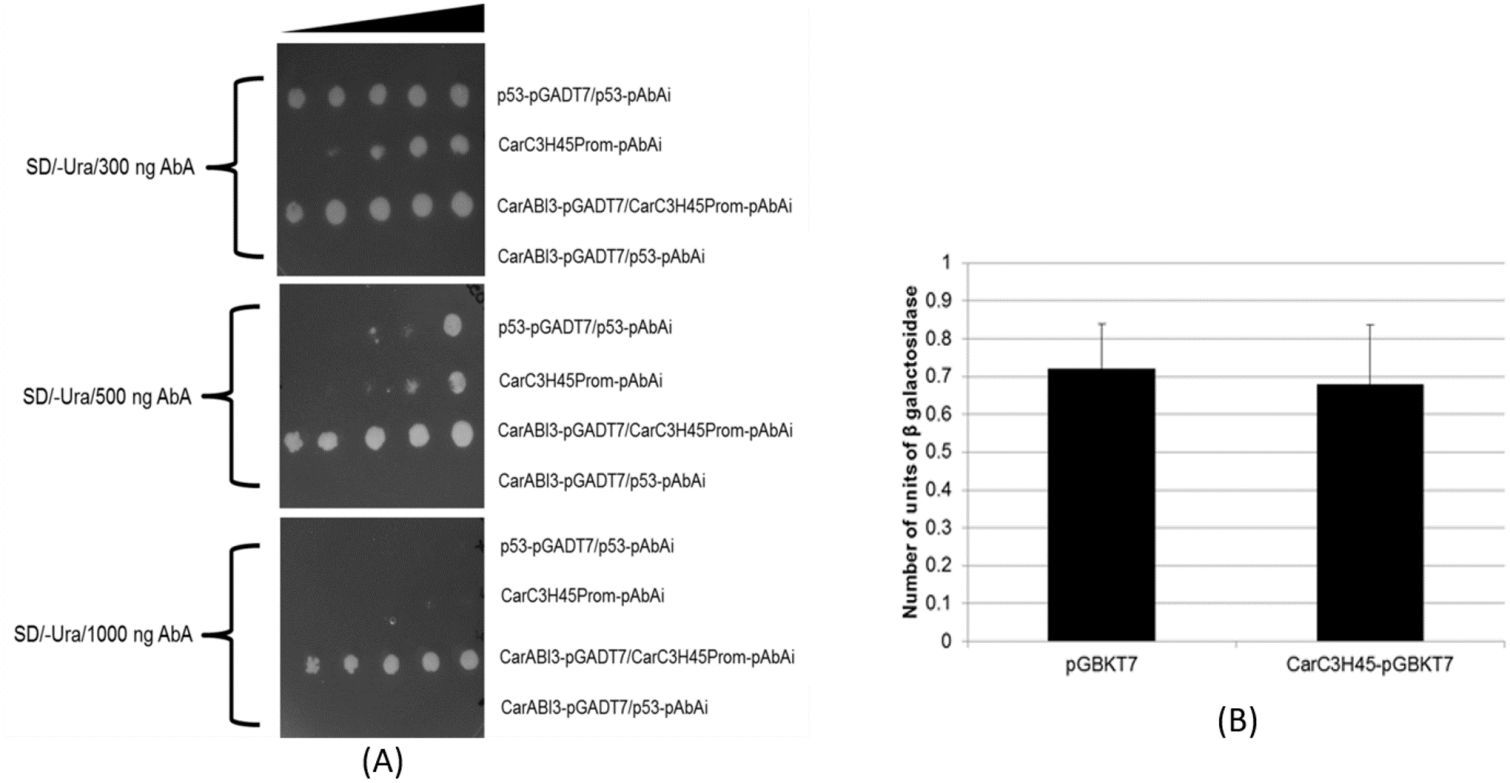
**(A)** Yeast one hybrid assay for determination of CarABI3-*CaC3H45Prom* interaction. p53-pGADT7/p53-pAbAi was used as positive control and CarABI3-pGADT7/p53-pAbAi was used as negative control. **(B)** Transactivation assay in yeast shows no significant transactivation activity for CarC3H45.

Transcription factors display transactivation activity which can be measured by yeast β-galactosidase assay. Therefore, the yeast β-galactosidase assay was carried out to determine the effect of CarC3H45 on the number of units of β-galactosidase enzyme produced in yeast cells. Full length CDS of CarC3H45 was cloned into pGBKT7 vector which contains a GAL4-DNA binding domain and used to carry out the assay. It was observed that there was no significant difference in the number of units of β-galactosidase produced in case of both negative control (pGBKT7) and for CarC3H45-pGBKT7 containing yeast cells (Fig 10B). The results suggested that CarC3H45 may not possess an activation domain that could activate gene transcription.

## Discussion

Genome wide analysis of gene families provides valuable insights into regulation of biological processes in plants and also serves as a foundation for identifying candidates for further characterisation of important genes. The *in silico* methods available today have made it possible to predict gene families on a genome wide level. Using these tools genome wide analysis of the CCCH Znf family in chickpea led to the prediction of 58 CCCH Zinc finger genes. Our observations revealed that the genome size does not determine the number of CCCH TFs reported for a species. The number of CCCH TFs identified in chickpea was higher than those predicted in *M*. *truncatula* [19] but lower than those reported in Arabidopsis [9], both of which have genome sizes smaller than chickpea. The number of CCCH motifs in each *CarC3H* gene was seen to range from one to six and even in other plants, as many as six CCCH motifs have been reported to be present in a single CCCH TF [9, 18, 19, 20]. The 58 CarC3H proteins were also seen to exhibit a wide range of isoelectric points (pI). The isoelectric point and charge of a protein are known to be important for its solubility, subcellular localization as well as interaction. There is a correlation between subcellular location and protein pI. For example, proteins in the cytoplasm possess an acidic pI (< 7.4), while those in the nucleus have a more neutral pI (7.4 <pI < 8.1) [40, 41]. The diversity in their lengths, motif patterns and isoelectric points (pI) suggested that the chickpea CCCH Zn finger genes are involved in a wide range of biological functions and may be regulating various aspects of chickpea plant development.

Phylogenetic analyses have been of paramount importance in the study of evolution of species [42]. The origin and course of evolution of a gene family can provide useful data about its functions and relative importance in a species [43, 44, 45]. Members of a clade were seen to display similarities in their structural organisations and functional domains. It was observed that while the numbers of introns and exons might vary amongst members of a clade, they had remarkably similar pattern of intron-exon arrangement. This could be indicative of exon shuffling during the course of evolution [46]. Presence of similar functional domains in the protein sequences of members belonging to a particular group further corroborates this assumption. Comparative phylogeny between CCCH TFs of chickpea, *M*. *truncatula* and Arabidopsis showed that the clustering was based on the presence of specific functional domains and not according to their plant species which implied that the CCCH TF family is highly conserved across the plant kingdom. In addition, the comparative phylogenetic analysis also revealed important CarC3H homologs in Arabidopsis. For example, CarC3H1 showed close homology with HUA1 in Arabidopsis, which has been reported to be involved in floral development [16]. Similarly, CarC3H17 (group VI, Fig 2) was found to be closely related to AtSZF1 which has been implicated in regulation of salt stress related genes [17]. CarC3H58 from group V of the phylogenetic tree (Fig 2) was found to be a close homolog of AtTZF1, a key regulator of ABA/sugar and GA responses in Arabidopsis [15] and CarC3H45 (group V) was found to be closely related to SOMNUS, one of the most extensively studied CCCH Znf proteins in Arabidopsis and implicated in seed development [39]. Overall analysis indicated that there were a number of CarC3H members which could have potentially vital roles in the process of chickpea development.

Synteny analysis with the genomes of other plants revealed that the *CarC3H* genes showed higher levels of similarity with *M*. *truncatula* genome as compared with *G*. *max*, *P*. *vulgaris* and Arabidopsis genomes. This reiterates the observations put forth after studying the evolution of chickpea plant. Both chickpea and *M*. *truncatula* belong to the galegoid clade of the Papillionoideae family while soybean belongs to the millettioid clade. The two clades separated about 54 million years (Myr) ago [47] and genome analysis of the galegoid species revealed that chickpea diverged from *M*. *truncatula* ~10–20 Myr ago. Therefore, *M*. *truncatula* is considered to be a closer relative of chickpea.

Various evolutionary forces act to determine the inheritance of advantageous elements to ensure propagation of species. Both positive (diversifying) and negative (purifying) selections play important roles in the evolution of gene families. Positive selection leads to high variability due to fixation of advantageous mutations in genes which in turn is responsible for species divergence. On the other hand, purifying selection is the default process of elimination of the unfit. The analysis of non-synonymous (Ka) and synonymous (Ks) substitutions in duplicated genes is an efficient method to study the evolution of important genes [48]. A Ka/Ks ratio of 1 indicates neutral selection while Ka/Ks > 1 indicates positive (diversifying) selection and Ka/Ks < 1 indicates negative (purifying) selection [29]. This method was used to determine evolutionary characteristics of the *CarC3H* gene family and it was observed that the duplication events in *CarC3H* genes are a result of purifying selection (Ka/Ks < 1) which means that in the course of evolution deleterious mutations have been eliminated to conserve their functions in chickpea. Genes under purifying selection are known to persist over long evolutionary time frames [49, 50]. Therefore, it may be assumed that the *CarC3H* gene family has a very important role in the development of the chickpea plant which has necessitated the conservation and propagation of its members. The presence of specific motifs in the protein sequences largely contribute towards determining their function. *In-silico* analysis of CarC3H proteins revealed the presence of a number of motifs in addition to the CCCH motif. All the three members of group III were found to contain KH type domain in their protein sequence. KH domains are known to bind RNA or ssDNA, and are found in proteins associated with transcriptional and translational regulation, along with other cellular processes [51]. Members of group VI and VII were seen to contain ankyrin repeat motifs which were first identified in the yeast cell cycle regulator Swi6/Cdc10 and the *Drosophila* signalling protein Notch [52]. The ankyrin repeat containing domains act as a scaffold for molecular interactions which are important for development of the numerous signalling pathways [53]. Members of group IX and XI in the phylogenetic tree were found to contain WD40 repeats and Zn finger RING motifs respectively. The WD40 repeat motifs and Zn finger RING domains are known to be involved in various important biological processes like signalling, cytoskeletal dynamics, protein trafficking, nuclear export and RNA processing. The WD40 repeats have been especially implicated in histone remodelling [54]and in flower development [55]. A number of CarC3H proteins such as CarC3H19, 34, 23, 12 and 32 contained RNA Recognition Motif (RRM) which are not only involved in RNA/DNA recognition but also in protein–protein interaction [56].

Digital expression analysis revealed the pattern of expression of *CarC3H* genes in different tissues of chickpea. Validation of this data through qRT PCR revealed that *CarC3H26* and *51* had higher expression during early stages of chickpea seed development while *CarC3H11* and *45* were specifically expressed in later stages. In addition, *CarC3H10* and *58* had higher expression in germinating seed tissues. Moreover, several genes were found to contain tissue specific motifs in their promoters, however no reliable correlation could be established between presence of the specific promoter motifs and expression of genes in those tissues. The data further endorsed the assumption that *CarC3H* genes were involved in a variety of regulatory roles. Detailed analysis of *CarC3H45*, a member of group V of the phylogenetic tree, showed that it was an intronless gene containing the plant specific unique arginine rich tandem zinc finger (RR-TZF) motif as was evident from the alignment with known AtTZFs [57, 58]. It has been observed that, ABA accumulation in developing seeds is low during the early stages, highest during the middle stage i.e. during accumulation of storage reserves and declines as the seed undergoes maturation drying. A number of studies involving analysis of mutants deficient in ABA responsiveness provide support for the hypothesis that the absence of, or insensitivity to, ABA during seed development results in the production of precociously germinating seeds [59, 60]. Expression analysis revealed that CarC3H45 had preferential expression in the later stage of seed development i.e. at 40 DAA while the expression was drastically reduced during seed germination. Application of exogenous ABA and GA to chickpea seeds showed that the levels of expression of CarC3H45 were increased during ABA treatment and reduced during GA treatment. Although the above data suggested probable regulation of this gene by ABA, this alone did not indicate a role for *CarC3H45* in regulation of dormancy.

In order to endorse the quantitative expression data, the promoter of *CarC3H45* was analysed for the presence of specific motifs which may indicate regulation by ABA. The RY repeat motif is known to be the binding site for B3 domain containing proteins such as ABI3 [61]. There exists a direct correlation between ABI3 and ABA since ABA is known to regulate dormancy through various signalling components, including three positive components, ABA-INSENSITIVE3 (ABI3), ABI4, and ABI5 [62]. The RY repeat motif was found to be present in the promoter region of *CarC3H45*. Yeast one hybrid assay showed that the CarABI3 protein could bind to the promoter of *CarC3H45* thereby conclusively establishing the role of this gene in regulation of chickpea seed dormancy. However, due to the absence of transactivation activity, it could potentially be designated as a transcriptional regulator whose probable interaction with other molecules needs to be investigated in order to establish the regulatory role of this important gene in chickpea.

## Methods

### Plant materials, growth conditions and stress treatments

Chickpea plants (*Cicer arietinum* cv. ICCV2, Kabuli type) were grown in the fields at NIPGR, India and used for tissue collection. Flowers were tagged on days of full anthesis and seeds were collected 10, 20, 30 ad 40 days after anthesis (DAA). The seeds were removed from their pods and immediately frozen in liquid nitrogen and stored at -80°C until further use. Flowers were collected from the field grown plants. Chickpea seedlings used for tissue-specific analysis were grown under control conditions (16hr/8hr light/dark photoperiod, 22±1°C/ 20±1°C day/night temperature, 65% relative humidity) in the growth chambers at the Plant Growth Facility, NIPGR, India. Leaves and roots were collected from 3 week old seedlings. For collection of germinating seedlings, the seeds were surface sterilised using 70% ethanol and imbibed overnight in water. They were spread on blotting paper for germination and tissue was collected at 24 hr, 48 hr and 72 hr intervals.

For various stress treatments, chickpea plants were grown in controlled conditions at the Plant Growth Facility and 3 week old seedlings were subjected to dehydration, desiccation, salinity and cold stress. Seedlings were subjected to dehydration stress by putting them in 1/2 strength MS medium supplemented with 20% PEG4000. The roots of seedlings were placed between blotting sheets to impart desiccation stress and salinity stress was given by putting the roots of the seedlings in 150mM NaCl solution. For cold stress, the seedlings in their pots were kept at 4^0^ C in the cold room for different time periods. Seedlings were subjected to the stress conditions for 0, 3, 6, 12 and 24 hrs and the 0hr samples were used as control.

### Genome wide prediction of CCCH zinc finger proteins in chickpea

The HMM profile for Zinc finger C-X_8_-C-X_5_-C-X_3_-H type and similar proteins was downloaded from Pfamdatabase (http://pfam.xfam.org/family/PF00642). The hmmsearch function of HMMER (v.3.1) program was used to search for the defined profile in the predicted proteins in the chickpea genome [23]. The predicted CCCH domain containing protein sequences were isolated and checked using SMART (http://smart.embl-heidelberg.de/smart/batch.pl) and Pfam (http://pfam.xfam.org/search) to confirm the presence of this domain. The sequences thus obtained were aligned using Clustal omega (http://www.ebi.ac.uk/Tools/msa/clustalo/) and any redundancy was manually removed.

### Prediction of genomic organisation and structural domains

Genomic organisation of the CCCH zinc finger genes was predicted using Gene Structure Display Server 2.0 (GSDS; http://gsds.cbi.pku.edu.cn/). The GFF3 file containing information about the intron-exon structure of *CarC3H* genes was used as input. Structural domains in protein sequences were predicted using ScanProsite (http://prosite.expasy.org/scanprosite/) which provided information about positions of different domains in the protein sequence. This information was used to draw visual representation of distribution of various domains in the amino acid sequences of proteins using DOG v. 2.0 (http://dog.biocuckoo.org/links.php). Conserved motifs in the amino acid sequences were predicted using the online tool Multiple Em for Motif Elicitation (MEME, http://meme-suite.org/tools/meme) with the following parameters: Motif discovery mode-Normal mode; Site distribution- Any number of repetitions; Number of motifs-10; Width of motifs- 6 to 50.

### Phylogenetic tree construction

Amino acid sequences of proteins were aligned using ClustalW and the alignment file was used to generate the phylogenetic tree using Neighbour-joining method on MEGA5.2 (http://www.megasoftware.net/). The parameters used for construction of phylogenetic tree were as follows: Statistical method- Neighbour-joining; Test of phylogeny- Bootstrap method with 1000 replications; Model/method- Poisson model with uniform rates of distribution; Gaps/missing data treatment- Pairwise deletion.

### Synteny analysis

Synteny analysis was carried out by mapping the gene sequences to genomes of chickpea, *Medicago truncatula*, soybean, *Phaseolus vulgaris* and Arabidopsis (Phytozome v10.0) using BlastN with an e-value cut-off of 1E-05 (ftp://ftp.ncbi.nlm.nih.gov/blast/executables/blast+/2.2.25/). The information was used to generate diagrams using Circos 0.67 [63].

### Chromosomal location, duplication analysis and calculation of Ka/Ks

The stand-alone version of Blast (ftp://ftp.ncbi.nlm.nih.gov/blast/executables/blast+/2.2.25/) was used to map the nucleotide sequences onto the kabuli chickpea genome [23] to determine the positions in the genome where the genes were located. The MapChart software (https://www.wageningenur.nl/en/show/Mapchart.htm) was then used to derive the diagrammatic representation of location of genes on the 8 linkage groups of chickpea. Tandem and segmental duplication events were identified based on the information available in Plant Genome Duplication Database (PGDD; http://chibba.agtec.uga.edu/duplication/). To calculate Ka/Ks for duplicated genes, the CDS and amino acid sequences of duplicated gene pairs were analysed on PAL2NAL software [64], http://www.bork.embl.de/pal2nal/) which uses the codeml program in PAML (Phylogenetic Analysis by Maximum Likelihood) to calculate Ka and Ks values. These values for each gene pair were obtained and manually divided to get Ka/Ks values.

### Digital gene expression analysis

The 454 reads for expression analysis in chickpea tissues- leaf, root, flower bud, pod and seed were retrieved from SRA (Sequence Read Archive) available under accession numbers SRX048833, SRX048832, SRX048834, SRX048835 [32] and SRX125162 [33], respectively. Short reads for chickpea root and shoot tissue under three stress conditions- desiccation, salinity and cold, were retrieved from SRA database available under accession number SRP034839 [65]. The reads were mapped onto the predicted gene models in *kabuli* chickpea genome [23] using BWA-MEM [66] for 454 reads and BWA [67] for Illumina reads. Mapped reads were extracted using SAM tools [68] and were used for calculating the RPKM (reads per kb per million mapped) values [69]. The RPKM values for *CarC3H* genes were utilized for generating the heat maps and k-means clustering using the MeV software [70].

### Quantitative real time PCR

Total RNA was isolated from different tissues using LiCl precipitation method as described by Pradhan et al. (2014). First strand cDNA was synthesized by reverse transcription from 3 μg of total RNA in 20 μl of reaction volume using AccuScript High Fidelity 1^st^ strand cDNA synthesis Kit (Agilent technologies, USA) as per manufacturers’ instructions. 5X dilutions of all cDNA samples were used for Real time PCR analysis. Gene specific primers were designed using PRIMER EXPRESS version 3.0 (Applied Biosystems, USA) with default parameters. Reactions were carried out in a final volume of 10 μl with 200 nM of each primer mixed with SYBR Green PCR master mix (Brilliant III Ultra-Fast SYBR Green QPCR Master Mix, Agilent technologies, USA) and 1μl of 5X diluted first strand cDNA, as per manufacturer’s instructions. The reaction was carried out in 96-well optical reaction plates (Applied Biosystems, USA), using Applied Biosystems’ ViiA7 Real Time PCR system and software (Applied Biosystems, USA). To normalize the variance among samples, *Elongation factor 1α* and *HSP90* were used as endogenous controls. Relative expression values were calculated after normalizing against the reference expression value (leaf in case of tissues and control in case of stressed samples). The values presented are the mean of the three biological replicates, each with three technical replicates. The error bars indicate standard deviation. All primers have been listed in S4 Table.

### Southern blotting

Genomic DNA was isolated as described by Doyle and Doyle [71]. About 10 μg of genomic DNA was digested with *Bam*HI, *Sac*I and *EcoR*I (NEB, USA). Digested DNA was separated on 0.8% (w/v) agarose gel, and transferred onto Hybond-N nylon membrane (Amersham Biosciences, UK) in 20x SSC. Briefly, the gel was run at 20-30V until it reached 2/3rd the distance. The gel was rinsed in autoclaved MQ. Depurination step was carried out by rinsing the gel in 0.125N HCl for 10–15 min. After washing the gel with MQ 2–3 times, the gel was rinsed in denaturing solution for 30 min with gentle shaking. Lastly, the gel was gently shaken in neutralization solution for 30 min. Transfer of DNA to membrane was carried out according to Sambrook’s protocol [72]. The membrane was washed in 2x SSC and UV-crosslinked. The blot was kept in hybridization bottle containing 10 ml Prehybridization buffer (0.1 M sodium phosphate buffer- pH-7.2, 10% SDS and 0.5M EDTA) and incubated at 60°C for 4–5 hrs. The radioisotope labelled probe was added to the hybridization bottle with the blot and allowed to hybridize at 60°C overnight. After hybridization, the blots were washed with 2X SSC, 0.1% SDS for 10 min at 60°C followed by washing with 1X SSC, 0.1% SDS for 10 min at room temperature. The membranes were exposed to the storage phosphorscreens (Amersham Biosciences, UK) for 30min to 1 hr. Images were acquired by scanning the membranes with Typhoon 9210 scanner (Amersham Biosciences, UK).

### Yeast one hybrid assay and transactivation assay

For yeast one hybrid assay, the promoter sequence of *CarC3H45* (about 1500 bp) was amplified from genomic DNA of chickpea and cloned into pAbAi vector between the *Sac*I and *Xho*I restriction sites. The ligated product was transformed into competent Y1HGold strain of yeast using Fast yeast transformation kits (G Biosciences), as per manufacturer’s instructions. pAbAi+Bait (*CarC3H45Prom*) (100 ng) was transformed into *S*. *cerevisaiae*Y1H Gold strain. About 100 μl of a 1/10 dilution and a 1/100 dilution of the transformation mixture were spread onto separate plates containing different concentrations of Aureobasidin to check for autoactivation. The pGADT7-*CarABI3* prey construct was then transformed into these competent cells and plated on SD/-Ura plates containing suitable amount of Aureobasidin and grown at 30°C for 2–3 days. Yeast colony PCR was carried out to confirm the presence of both bait and prey DNA sequences. The Y1HGold competent cells were also transformed with p53-pAbAi/p53-pGADT7 constructs, to be used as positive control and *CarABI3-*pGADT7/p53-pAbAi, to be used as negative control. Yeast colony PCR was performed and positive colonies were inoculated in 5 ml of YPDA medium and grown overnight at 30°C. The overnight cultures were diluted (3X) in 0.9% NaCl solution. Serial dilutions of 5X were prepared successively from each and spotted onto SD/-Ura/AbA plates. The plates were observed after 2-3days.

To perform transactivation assay, constructs containing the full CDS of *CarC3H45* in pGBKT7 vector were transformed into competent yeast cells (strain AH109) using Fast yeast transformation kits (G Biosciences), as per manufacturer’s instructions. About 80μl of the transformed yeast cells were plated on SD/-Trp plates and grown at 30^0^ C for 2–3 days to obtain colonies. A single positive colony was picked and grown overnight in 5ml of SD/-Trp liquid medium. The culture was vortexed to disperse clumped yeast cells and 2 ml of this culture was added to 8 ml of YPDA broth. The culture was incubated at 30^0^ C with shaking (220–240 rpm) till the cells were in mid-log phase (OD_600_ = 0.5–0.8) and the exact OD was recorded. ONPG was dissolved at a concentration of 4mg/ml in Z buffer with shaking for 1-2hr. 1.5ml of the secondary culture was taken in three replicates, centrifuged at 14,000 rpm for 1 min. and supernatant was removed. 1.5ml of Z buffer was added to each tube and cells were resuspended by vortexing. The cells were centrifuged as before and supernatants were removed. The pellets were resuspended in 300 μl of Z buffer thereby giving a final concentration factor of 5. From this, 100 μl was transferred into a fresh 1.5 ml eppendorf tube and the tubes were placed in liquid nitrogen for 30 secs and then in 37^0^ C water bath for 30 sec. This was repeated another three times. To 100 ml of Z buffer, 0.27 ml of β-mercaptoethanol was added and 0.7 ml of this was added to each tube, including a blank tube containing 100 μl of Z buffer. 160 μl of ONPG in Z buffer was added to each tube and tubes were placed at 30^0^ C in an incubator and timer was started. As soon as yellow colour developed, 0.4 ml of 1M Na_2_CO_3_ solution was added to all tubes and elapsed time was recorded in minutes. Tubes were centrifuged at 14,000 rpm and supernatants were collected. Absorbance was measured for the samples at 420nm and β-galactosidase units were calculated as follows:

β−galactosidase  units = 1,000 x OD420 /(t x V x OD600)

where: t = elapsed time (in min) of incubation; V = 0.1 ml x concentration factor (5 in this case); OD_600_ = A_600_ of 1 ml of culture

## Supporting information

S1 Table Sequence information for *CarC3H* genes. (XLSX)

S2 Table Description of CCCH motifs present in the CarC3H sequences. (DOCX)

S3 Table Details of motifs found in the promoter sequences of *CarC3H* genes. (DOCX)

S4 Table Primer sequences used for real time qRT-PCR. (DOCX)

## References

[1] Li J, Jia D and Chen X. HUA1, a regulator of stamen and carpel identities in Arabidopsis, codes for a nuclear RNA-binding protein. Plant Cell. 2001;13, 2269–2281 11595801 10.1105/tpc.010201 PMC139158

[2] Gao G, Guo X and Goff SP. Inhibition of retroviral RNA production by ZAP, a CCCH-type zinc finger protein. Science. 2002;297, 1703–1706 doi: 10.1126/science.1074276 12215647 10.1126/science.1074276

[3] Kong Z, Li M, Yang W, Xu W and Xue Y. A novel nuclear-localized CCCH-type zinc finger protein, OsDOS, is involved in delaying leaf senescence in rice. Plant Physiol. 2006;141, 1376–1388 doi: 10.1104/pp.106.082941 16778011 10.1104/pp.106.082941 PMC1533915

[4] Guo YH, Yu YP, Wang D, Wu CA, Yang GD, Huang JG et al GhZFP1, a novel CCCH-type zinc finger protein from cotton, enhances salt stress tolerance and fungal disease resistance in transgenic tobacco by interacting with GZIRD21A and GZIPR5. New Phytol. 2009;183, 62–75 doi: 10.1111/j.1469-8137.2009.02838.x 19402879 10.1111/j.1469-8137.2009.02838.x

[5] Wang L, Xu Y, Zhang C, Ma Q, Joo SH, Kim SK et al OsLIC, a novel CCCH-type zinc finger protein with transcription activation, mediates rice architecture via brassinosteroids signaling. PloS One. 2008;3(10), e3521–e3521 doi: 10.1371/journal.pone.0003521 18953406 10.1371/journal.pone.0003521 PMC2567845

[6] Hall TM. Multiple modes of RNA recognition by zinc finger proteins. Curr Opin Struct Biol. 2005;15, 367–373 doi: 10.1016/j.sbi.2005.04.004 15963892 10.1016/j.sbi.2005.04.004

[7] Berg JM and Shi Y. The galvanization of biology: a growing appreciation for the roles of zinc. Science 1996;271: 1081–1085 8599083 10.1126/science.271.5252.1081

[8] Takatsuji H. Zinc-finger transcription factors in plants. Cell Mol Life Sci. 1998;54, 582–596 doi: 10.1007/s000180050186 9676577 10.1007/s000180050186 PMC11147231

[9] Wang D, Guo Y, Wu C, Yang G, Li Y and Zheng C.Genome-wide analysis of CCCH zinc finger family in Arabidopsis and rice. BMC genomics. 2008;9(1), 44 18221561 10.1186/1471-2164-9-44 PMC2267713

[10] Bai C and Tolias PP. Cleavage of RNA hairpins mediated by a developmentally regulated CCCH zinc finger protein. Mol Cell Biol. 1996;16, 6661–6667 8943320 10.1128/mcb.16.12.6661 PMC231668

[11] Lai WS, Carballo E, Strum JR, Kennington EA, Phillips RS and Blackshear PJ. Evidence that tristetraprolin binds to AU-rich elements and promotes the deadenylation and destabilization of tumor necrosis factor alpha mRNA. Mol Cell Biol. 1999;19, 4311–4323 10330172 10.1128/mcb.19.6.4311 PMC104391

[12] Lai WS, Carballo E, Thorn JM, Kennington EA and Blackshear PJ. Interactions of CCCH zinc finger proteins with mRNA binding of tristetraprolin-related zinc finger proteins to Au-rich elements and destabilization of mRNA. Journal of Biological Chemistry. 2000;275(23), 17827–17837 doi: 10.1074/jbc.M001696200 10751406 10.1074/jbc.M001696200

[13] Hurt JA, Obar RA, Zhai B, Farny NG, Gygi SP and Silver PA. A conserved CCCH-type zinc finger protein regulates mRNA nuclear adenylation and export. The Journal of cell biology. 2009;185(2), 265–277 doi: 10.1083/jcb.200811072 19364924 10.1083/jcb.200811072 PMC2700372

[14] Li Z and Thomas TL. PEI1, an embryo-specific zinc finger protein gene required for heart-stage embryo formation in Arabidopsis. Plant Cell. 1998;10, 383–398 9501112 10.1105/tpc.10.3.383 PMC143998

[15] Lin PC, Pomeranz MC, Jikumaru Y, Kang SG, Hah C, Fujioka S et al The Arabidopsis tandem zinc finger protein AtTZF1 affects ABA and GA-mediated growth, stress and gene expression responses. Plant J. 2011;65 253–268. doi: 10.1111/j.1365-313X.2010.04419.x 21223390 10.1111/j.1365-313X.2010.04419.x

[16] Cheng Y, Kato N, Wang W, Li J and Chen X. Two RNA binding proteins, HEN4 and HUA1, act in the processing of AGAMOUS pre-mRNA in Arabidopsis thaliana. Developmental cell. 2003;4(1), 53–66. 12530963 10.1016/s1534-5807(02)00399-4 PMC5135010

[17] Sun J, Jiang H, Xu Y, Li H, Wu X, Xie Q et al The CCCH-type zinc finger proteins AtSZF1 and AtSZF2 regulate salt stress responses in Arabidopsis. Plant Cell Physiol. 2007;48, 1148–1158. doi: 10.1093/pcp/pcm088 17609218 10.1093/pcp/pcm088

[18] Peng X, Zhao Y, Cao J, Zhang W, Jiang H, Li X et al CCCH-type zinc finger family in maize: genome-wide identification, classification and expression profiling under abscisic acid and drought treatments. PLoS One. 2012;7, e40120 doi: 10.1371/journal.pone.0040120 22792223 10.1371/journal.pone.0040120 PMC3391233

[19] Zhang C, Zhang H, Zhao Y, Jiang H, Zhu S, Cheng B et al Genome wide analysis of the CCCH zinc finger gene family in Medicago truncatula. Plant Cell Rep. 2013;32, 1543–1555. doi: 10.1007/s00299-013-1466-6 23749175 10.1007/s00299-013-1466-6

[20] Liu S, Khan MRG, Li Y, Zhang J and Hu C. Comprehensive analysis of CCCH-type zinc finger gene family in citrus (Clementine mandarin) by genome-wide characterization. Molecular Genetics and Genomics. 2014;289(5), 855–872. doi: 10.1007/s00438-014-0858-9 24820208 10.1007/s00438-014-0858-9

[21] Wang XL, Zhong Y and Cheng ZMM. Evolution and Expression Analysis of the CCCH Zinc Finger Gene Family in Vitis vinifera. The Plant Genome. 2014;7(3).

[22] Jain M, Misra G, Patel RK, Priya P, Jhanwar S, Khan AW et al A draft genome sequence of the pulse crop chickpea (Cicer arietinum L). The Plant Journal. 2013;74, 715–729 doi: 10.1111/tpj.12173 23489434 10.1111/tpj.12173

[23] Varshney RK, Song C, Saxena RK, Azam S, Yu S, Sharpe AG et. al Draft genome sequence of chickpea (Cicer arietinum) provides a resource for trait improvement. Nat Biotechnol. 2013;31,240–246 doi: 10.1038/nbt.2491 23354103 10.1038/nbt.2491

[24] Blackshear PJ. Tristetraprolin and other CCCH tandem zinc-finger proteins in the regulation of mRNA turnover. Biochemical Society Transactions. 2002;30(Pt 6), 945–952. 12440952 10.1042/bst0300945

[25] Kishore S, Luber S and Zavolan M. Deciphering the role of RNA-binding proteins in the post-transcriptional control of gene expression. Briefings in functional genomics. 2010;9(5–6), 391–404 doi: 10.1093/bfgp/elq028 21127008 10.1093/bfgp/elq028 PMC3080770

[26] Duran C, Edwards D and Batley J. Genetic maps and the use of synteny. In Plant Genomics. Humana Press. 2009; pp 41–55. 10.1007/978-1-59745-427-8_3 19347649

[27] Choi HK, Mun J, Kim D, Zhu H, Baek J, Mudge J et. al Estimating genome conservation between crop and model legume species. Proc Natl Acad Sci USA. 2004;101(43), 15289–15294. doi: 10.1073/pnas.0402251101 15489274 10.1073/pnas.0402251101 PMC524433

[28] Sharp AJ, Locke DP, McGrath SD, Cheng Z, Bailey JA, Vallente RU et. al Segmental duplications and copy-number variation in the human genome. The American Journal of Human Genetics. 2005;77(1), 78–88. doi: 10.1086/431652 15918152 10.1086/431652 PMC1226196

[29] Wagner A. Selection and gene duplication: a view from the genome. Genome Biol. 2002;3(5), 1012. 10.1186/gb-2002-3-5-reviews1012 PMC139360 12049669

[30] Li WH and Gojobori T Rapid evolution of goat and sheep globin genes following gene duplication. Mol Biol Evol. 1983;1, 94–108. 6599963 10.1093/oxfordjournals.molbev.a040306

[31] Hughes AL and Nei M Pattern of nucleotide substitution at major histocompatibility complex class I loci reveals over dominant selection. Nature. 1998;335, 167–170 10.1038/335167a0 3412472

[32] Garg R, Patel RK, Jhanwar S, Priya P, Bhattacharjee A, Yadav G et. al Gene discovery and tissue-specific transcriptome analysis in chickpea with massively parallel pyrosequencing and web resource development. Plant Physiol. 2011;156,1661–1678. doi: 10.1104/pp.111.178616 21653784 10.1104/pp.111.178616 PMC3149962

[33] Pradhan S, Bandhiwal N, Shah N, Kant C, Gaur R and Bhatia S. Global transcriptome analysis of developing chickpea (Cicer arietinum L.) seeds. Front Plant Sci. 2014;5, 1–14. 10.3389/fpls.2014.00698 PMC4267183 25566273

[34] Kim DH, Yamaguchi S, Lim S, Oh E, Park J, Hanada A et al SOMNUS, a CCCH-type zinc finger protein in Arabidopsis, negatively regulates light-dependent seed germination downstream of PIL5. Plant Cell. 2008b;20:1260–1277. 18487351 10.1105/tpc.108.058859 PMC2438461

[35] Lin PC, Pomeranz MC, Jikumaru Y, Kang SG, Hah C, Fujioka S et al The Arabidopsis tandem zinc finger protein AtTZF1 affects ABA and GA-mediated growth, stress and gene expression responses. Plant J. 2011;65: 253–268. doi: 10.1111/j.1365-313X.2010.04419.x 21223390 10.1111/j.1365-313X.2010.04419.x

[36] Bogamuwa SP and Jang JC. The Arabidopsis tandem CCCH zinc finger proteins AtTZF4, 5 and 6 are involved in light, abscisic acid and gibberellic acid mediated regulation of seed germination. Plant Cell Environ. 2013;(36): 1507–1519. 23421766 10.1111/pce.12084

[37] Carranco R, Chandrasekharan M, Townsend J and Hall T. Interaction of PvALF and VP1 B3 domains with the β-phaseolin promoter. Plant molecular biology 2004;55(2): 221–237. doi: 10.1007/s11103-004-0512-8 15604677 10.1007/s11103-004-0512-8

[38] Gutierrez L, Van Wuytswinkel O, Castelain M and Bellini C. Combined networks regulating seed maturation. Trends in plant science. 2007;12(7): 294–300. doi: 10.1016/j.tplants.2007.06.003 17588801 10.1016/j.tplants.2007.06.003

[39] Ouwerkerk P B and Meijer A H. Yeast One-Hybrid Screening for DNA-Protein Interactions. Current protocols in molecular biology. 2001;12–12. 10.1002/0471142727.mb1212s55 18265084

[40] Andrade MA, O'Donoghue SI, and Rost B. Adaptation of protein surfaces to subcellular location. J Mol Biol. 1998;276(2), 517–25 doi: 10.1006/jmbi.1997.1498 9512720 10.1006/jmbi.1997.1498

[41] Nandi S, Mehra N, Lynn AM and Bhattacharya A. Comparison of theoretical proteomes: identification of COGs with conserved and variable pI within the multimodal pI distribution. BMC Genomics. 2005;6, 116 doi: 10.1186/1471-2164-6-116 16150155 10.1186/1471-2164-6-116 PMC1249567

[42] Huson DH and Bryant D. Application of phylogenetic networks in evolutionary studies. Mol Biol Evol. 2006;23, 254–267 doi: 10.1093/molbev/msj030 16221896 10.1093/molbev/msj030

[43] Nam J, Ma H and Nei M. Antiquity and evolution of the MADS-box gene family controlling flower development in plants. Molecular biology and evolution. 2003;20(9), 1435–1447. doi: 10.1093/molbev/msg152 12777513 10.1093/molbev/msg152

[44] Moore RC and Purugganan MD. The evolutionary dynamics of plant duplicate genes. Current opinion in plant biology. 2005;8(2), 122–128. doi: 10.1016/j.pbi.2004.12.001 15752990 10.1016/j.pbi.2004.12.001

[45] Tang H, Bowers JE, Wang X, Ming R, Alam M and Paterson AH. Synteny and collinearity in plant genomes. Science. 2008;320(5875), 486–488. doi: 10.1126/science.1153917 18436778 10.1126/science.1153917

[46] Kolkman JA and Stemmer WP. Directed evolution of proteins by exon shuffling. Nature biotechnology. 2001;19(5), 423–428 doi: 10.1038/88084 11329010 10.1038/88084

[47] Lavin M, Herendeen PS and Wojciechowski MF. Evolutionary rates analysis of Leguminosae implicates a rapid diversification of lineages during the tertiary. Syst. Biol. 2005;54,575–594. doi: 10.1080/10635150590947131 16085576 10.1080/10635150590947131

[48] Hanada K, Shiu SH and Li WH. The Nonsynonymous/synonymous substitution rate ratio versus the radical/conservative replacement rate ratio in the evolution of mammalian genes. Mol. Biol. Evol. 2007;24, 2235–2241 doi: 10.1093/molbev/msm152 17652332 10.1093/molbev/msm152

[49] Lynch M and Conery JS. The evolutionary fate and consequences of duplicate genes. Science. 2000;290(5494), 1151–1155 11073452 10.1126/science.290.5494.1151

[50] Tuskan GA, Difazio S, Jansson S, Bohlmann J, Grigoriev I, Hellsten U et. al The genome of black cottonwood, Populus trichocarpa (Torr.and Gray). Science. 2006;313(5793), 1596–1604. doi: 10.1126/science.1128691 16973872 10.1126/science.1128691

[51] Valverde R, Edwards L and Regan L. Structure and function of KH domains. FEBS journal. 2008;275(11), 2712–2726. doi: 10.1111/j.1742-4658.2008.06411.x 18422648 10.1111/j.1742-4658.2008.06411.x

[52] Breeden L and Nasmyth K. Cell cycle control of the yeast HO gene: cis-and trans-acting regulators. Cell. 1987;48(3), 389–397. 3542227 10.1016/0092-8674(87)90190-5

[53] Marcotte EM, Pellegrini M, Ng HL, Rice DW, Yeates TO and Eisenberg D. Detecting protein function and protein-protein interactions from genome sequences. Science. 1999;285(5428), 751–753. 10427000 10.1126/science.285.5428.751

[54] Couture JF, Collazo E and Trievel RC. Molecular recognition of histone H3 by the WD40 protein WDR5. Nat. Struct. Mol. Biol. 2006;13,698–703. doi: 10.1038/nsmb1116 16829960 10.1038/nsmb1116

[55] Van Nocker S and Ludwig P. The WD-repeat protein superfamily in Arabidopsis: conservation and divergence in structure and function. BMC genomics. 2003;4(1), 50 doi: 10.1186/1471-2164-4-50 14672542 10.1186/1471-2164-4-50 PMC317288

[56] Cléry A, Blatter M and Allain FH. RNA recognition motifs: boring? Not quite. Current opinion in structural biology. 2008;18(3), 290–298. doi: 10.1016/j.sbi.2008.04.002 18515081 10.1016/j.sbi.2008.04.002

[57] Pomeranz M C, Hah C, Lin P C, Kang S G, Finer J J, Blackshear P J et al The Arabidopsis tandem zinc finger protein AtTZF1 traffics between the nucleus and cytoplasmic foci and binds both DNA and RNA. Plant Physiol. 2010;152(1): 151–165. doi: 10.1104/pp.109.145656 19897605 10.1104/pp.109.145656 PMC2799353

[58] Bogamuwa SP and Jang JC. Tandem CCCH Zinc finger proteins in plant growth, development, and stress response. Plant and Cell Physiology. 2014;pcu074. 10.1093/pcp/pcu074 24850834

[59] Koornneef M and Karssen CM. 12 Seed dormancy and germination. Cold Spring Harbor Monograph Archive. 1994;(27): 313–334.

[60] McCarty D R. Genetic control and integration of maturation and germination pathways in seed development. Annual review of plant biology. 1995;46(1): 71–93.

[61] Reidt W, Wohlfarth T, Ellerström M, Czihal A, Tewes A, Ezcurra I et al Gene regulation during late embryogenesis: the RY motif of maturation-specific gene promoters is a direct target of the FUS3 gene product. The Plant Journal. 2000;21(5): 401–408. 10758492 10.1046/j.1365-313x.2000.00686.x

[62] Söderman E M, Brocard I M, Lynch T J and Finkelstein R R. Regulation and function of the Arabidopsis ABA-insensitive4 gene in seed and abscisic acid response signaling networks. Plant Physiol. 2000;124(4): 1752–1765. 11115891 10.1104/pp.124.4.1752 PMC59872

[63] Krzywinski M, Schein J, Birol I, Connors J, Gascoyne R, Horsman D et al Circos: an information aesthetic for comparative genomics. Genome Res. 2009;19, 1639–1645 doi: 10.1101/gr.092759.109 19541911 10.1101/gr.092759.109 PMC2752132

[64] Suyama M, Torrents D and Bork P. PAL2NAL: robust conversion of protein sequence alignments into the corresponding codon alignments. Nucleic acids research. 2006;34 (suppl 2), W609–W612. 16845082 10.1093/nar/gkl315 PMC1538804

[65] Garg R, Bhattacharjee A and Jain M. Genome-scale transcriptomic insights into molecular aspects of abiotic stress responses in chickpea. Plant Mol Biol Rep. 2014.

[66] Li H. Aligning sequence reads, clone sequences and assembly contigs with BWA-MEM. 2013;(http://arXiv.org/abs/1303.3997v2)

[67] Li H and Durbin R. Fast and accurate short read alignment with Burrows-Wheeler transform. Bioinformatics. 2009;25:1754–60. doi: 10.1093/bioinformatics/btp324 19451168 10.1093/bioinformatics/btp324 PMC2705234

[68] Li H, Handsaker B, Wysoker A, Fennell T, Ruan J, Homer N et al The Sequence alignment/map (SAM) format and SAM tools. Bioinformatics. 2009;25:2078–9. doi: 10.1093/bioinformatics/btp352 19505943 10.1093/bioinformatics/btp352 PMC2723002

[69] Mortazavi A, Williams BA, McCue K, Schaeffer L and Wold B Mapping and quantifying mammalian transcriptomes by RNA-Seq. Nat Methods. 2008;5(7):621–8. doi: 10.1038/nmeth.1226 18516045 10.1038/nmeth.1226 PMC13303166

[70] Saeed AI, Bhagabati NK, Braisted JC, Liang W, Sharov V, Howe EA et al TM4 microarray software suite. Methods Enzymol. 2006;411:134–93. doi: 10.1016/S0076-6879(06)11009-5 16939790 10.1016/S0076-6879(06)11009-5

[71] Doyle J. J., and Doyle J. L. A rapid DNA isolation procedure for small quantities of fresh leaf tissue. Phytochem Bull. 1987;19, 11–15

[72] Sambrook J, Russell DW. Molecular cloning: a laboratory manual. New York Cold Spring Harbor Laboratory (2001)

