# Supplementary material for: Genome-wide analysis of the CCCH zinc finger family identifies tissue specific and stress responsive candidates in chickpea (Cicer arietinum L.)
Source: PLoS One. 2017 Jul 12;12(7):e0180469. doi: 10.1371/journal.pone.0180469 (PMC5507508; doi:10.1371/journal.pone.0180469)
Supplement: S2 Table — (DOCX) [file pone.0180469.s002.docx]

**S2 Table. Description of CCCH motifs present in the CarC3H sequences**

| **Gene Name** | **Number of CCCH motifs** | **CCCH motifs** | **Type of CCCH motif** |
| --- | --- | --- | --- |
| CarC3H1 | 6 | CSHYMLTRTCKFGESCKFDH; CPYFLKTKKCKFGSRCKFNH; CAFYLKTGLCKYGAACKFHH; CPFYMKTGSCKFGATCRYNH; CDYYMKTGICKYGERCKYHH; CPYYLKTGTCKFGVTCKFDH | 853; 853; 853; 853; 853; 853 |
| CarC3H2 | 4 | CKFFAHGACLKGEHCEFSH; CTFYQKGVCAYGSRCRYDH; CSFAAAGNCPRGEQCPHVH; CKHFDFGDGNCPFGTSCFYKH | 853; 753; 753; 953 |
| CarC3H3 | 2 | CFQWQAGKCSRYPCPFLH; CTYWIQGNCSYGERCKFLH | 743; 753 |
| CarC3H4 | 3 | CRHYMNGRCHEGDKCNFSH; CTHFARHSCMKGDDCPFDH; CSNFLSKGSCYRGDACLFSH | 753; 753; 853 |
| CarC3H5 | 2 | CAYWLAGRCNRNPCRFLH; CKYWVNGNCVHGDRCRYLH | 743; 753 |
| CarC3H6 | 5 | CSFYMKTGSCKFGFNCKFNH; CKYYQRSGGCKFGKACKFNH; CPYYMRTGSCKFGSNCRFNH; CSFFLKTGDCKFKSNCKFHH; CSHYSRYGICKFGPACRFDH | 853; 853; 853; 853; 853 |
| CarC3H7 | 2 | CPFHLKTGACRFGERCSRVH; CGEYMKSGYKTCSHGTACNFIH | 853; 10-5-3 |
| CarC3H8 | 1 | CDFFAKGWCIRGSSCSFLH | 753 |
| CarC3H9 | 4 | CTYYLRTGFCGFGSRCRFNH; CQYYMRTRSCKFGASCKYHH; CSYFAKTGQCKFGATCKFDH; CTHYTQRGVCKFGPTCKFDH | 853; 853; 853; 853 |
| CarC3H10 | 2 | CRRFMQGVCFLGPKCNYAH; CRLFLRNKHCSYGHTCRFLH | 853; 853 |
| CarC3H11 | 2 | CSEYRRGSCNRGDTCEFAH; CKDGKNCKRKVCFFAH | 753; 543 |
| CarC3H12 | 2 | CPFYFKIGACRHGDRCSRLH; CRQYEENTCNRGGYCNFMH | 853; 753 |
| CarC3H13 | 1 | CFDFLRRKCYRGASCRFAH | 753 |
| CarC3H14 | 1 | CRDFEERGFCLRGDMCPMEH | 853 |
| CarC3H15 | 1 | CRAFQRGDCTRGAGCKFSH | 753 |
| CarC3H16 | 1 | CVPCPEFRKGSCSKGDLCEYAH | 10-5-3 |
| CarC3H17 | 2 | CPEFRKGSCQKGDSCEYAH; CKDETGCNRKVCFFAH | 753; 543 |
| CarC3H18 | 5 | CLYYLRTGMCGYGSNCRYNH; CEYFLKTGTCKYGSTCKYHH; CPYYMRTGSCKFGVACKFHH; CKYFMSTGTCKYGSDCKFHH; CSYYRLYGVCKFGPTCKFDH | 853; 853; 853; 853; 853 |
| CarC3H19 | 1 | CHYFRKGFCKHGSNCRYIH | 753 |
| CarC3H20 | 5 | CSYYIRTGLCRFGATCRFNH; CRFNHPPNRKLYYLKTGTCKFGATCRFHH; CAYYLRTGQCKFANTCKFHH; CQFYMKTGDCKFGAVCRFHH; CVFYSRYGICKFGPSCKFDH | 853; 17-5-3; 853; 853; 853 |
| CarC3H21 | 1 | CPDFRKGACRRGDMCEYAH | 753 |
| CarC3H22 | 3 | CTKFFSTSGCPFGESCHFLH; CNKFNTAEGCKFGDKCHFAH; CENFTKGSCTFGDRCHFAH | 853; 853; 753 |
| CarC3H23 | 2 | CPFYFKIGACRHGDRCSRLH; CRQYEENVCNRGGYCNFMH | 853; 753 |
| CarC3H24 | 1 | CKDYKETGYCGYGDSCKFMH | 853 |
| CarC3H25 | 5 | CSYYIRTGLCRFGATCRFNH; CRFNHPPNANLFYLKTGTCKFGATCKFHH; CSYYLRTGECKFGNTCKFHH; CQFYMKTGDCKFGAVCRFHH; CVFYSRYGICKFGPSCKFDH | 853; 17-5-3; 853; 853; 853 |
| CarC3H26 | 1 | CLHFVNKGFCRFGDSCKYFH | 853 |
| CarC3H27 | 4 | CVYYMRTGFCGYGGRCRFNH; CSYYLKTGQCKFGVTCKFHH; CQYYLRTGDCKFGLACRYHH; CSFYLQNGHCKFGSSCKFDH | 853; 853; 853; 853 |
| CarC3H28 | 2 | CNKWQETGTCPYGDHCQFAH; CRMVLAGVVCPYGHRCHFRH | 853; 853 |
| CarC3H29 | 3 | CTKFFSTSGCPFGEGCHFLH; CNKFNTAEGCKFGDKCHFAH; CENFTKGSCTFGEKCHFAH | 853; 853; 753 |
| CarC3H30 | 2 | CEFFKVGQCAKGFKCKFSH; CKHFLDAVERKQYGWFWACPNGGKNCHYRH | 753; 17-6-3 |
| CarC3H31 | 3 | CTKFFSIAGCPFGEGCHFLH; CNKFNSAEGCKFGDKCHFAH; CENFAKGSCTFGERCHFAH | 853; 853; 753 |
| CarC3H32 | 2 | CPFYFKIGACRHGDRCSRLH; CRQYEENSCNRGGYCNFMH | 853; 753 |
| CarC3H33 | 4 | CQFFTRFGKCNKDGGKCPYIH; CSYFLQGLCSNKNCPYRH; CEGFLKGFCADGNECRKKH; CPSFEATGTCTQGTKCKLHH | 863; 743; 753; 853 |
| CarC3H34 | 1 | CLYFARGYCKNGTSCRFLH | 753 |
| CarC3H35 | 4 | CKFHARGVCLKGEQCDFSH; CSYYQKGSCAYGSRCRYKH; CKYAAANCPNRDRCTRIH; CKHFSGGNGNCPFGAGCFYKH | 753; 753; 653; 953 |
| CarC3H36 | 1 | CPDFRKGACRRGDMCEYAH | 753 |
| CarC3H37 | 1 | CPEFRKGSCSKGDGCEYAH | 753 |
| CarC3H38 | 5 | CSFYLKTGTCKFGFNCKFNH; CKYYSRSGGCKFGNDCKFNH; CPYYMRTGSCKFGSSCKFNH; CSFFLKTGDCKFKSHCKFHH; CTHYSRYGICKFGPACKFDH | 853; 853; 853; 853; 853 |
| CarC3H39 | 1 | CSFYIRGECTRGAECPYRH | 753 |
| CarC3H40 | 3 | CRDFPAGKCRRGSLCNFLH; CINFAKGRCRMGASCKFVH; CKFFANGNCRNGKYCRFAH | 753; 753; 753 |
| CarC3H41 | 2 | CPDFRKGSCKKGDSCEFAH; CKDGTSCRRRVCFFAH | 753; 543 |
| CarC3H42 | 3 | CRDFAVGNCRRGSHCHFLH; CINFAKGRCRTGESCKYVH; CKFFAFGNCRNGKDCRFSH | 753; 753; 753 |
| CarC3H43 | 3 | CVYFLASPLTCKKGNECEYRH; CWYWLNGNCLNPKCSFRH; CIFFQKGFCLKGDRCAFSH | 953; 743; 753 |
| CarC3H44 | 1 | CRAWEESGNCRYNSKCQVRSRH | 855 |
| CarC3H45 | 1 | CPDYRRGNCERGDACEFSH; CKDGKNCKRKICFFAH | 753; 543 |
| CarC3H46 | 1 | CRNFQRGSCSYGERCRYLH | 753 |
| CarC3H47 | 1 | CSYFRKHATCHHGDACRYAH | 853 |
| CarC3H48 | 1 | CHYFNKGFCKNGNYCKYSH | 753 |
| CarC3H49 | 2 | CFKFVSSGSCPRGEQCNYRH; CFDFLNKGKCERGPDCSFRH | 853; 853 |
| CarC3H50 | 4 | CIYYLRTGFCGYGSRCRFNH; CQYYMRTGSCKFGASCKYHH; CHHYMKTGECKFGSSCRYHH; CTHYAQRGICKFGPACKFDH | 853; 853; 853; 853 |
| CarC3H51 | 3 | CVYFLASLTCKKGAECEYRH; CWFWLAGNCLNPTCAFRH; CFYFFNGFCNKGNRCSFLH | 853; 743; 753 |
| CarC3H52 | 3 | CVKFKLGSCRNGENCNFAH; CKKFYNGEECPYGDKCNFLH; CLKWEHTGHCPFGDDCHFAH | 753; 853; 853 |
| CarC3H53 | 3 | CCKFRVGTCPYVTNCNFAH; CKKFYTEEGCPYGESCTFLH; CNKWEMTGYCPFGNKCHFAH | 753; 853; 853 |
| CarC3H54 | 1 | CRHFARGRCYFGDNCKFLH | 753 |
| CarC3H55 | 2 | CHYWLAGRCNRNPCRFLH; CKYWMDENCVYGDHCRYLH | 743; 753 |
| CarC3H56 | 1 | CPEFRKGTCRRGDMCEYAH | 753 |
| CarC3H57 | 1 | CLYFARGFCKNGATCKFVH | 753 |
| CarC3H58 | 3 | CDHFRMYEFKIRRCARGRSHDWTECPYAH; CPDFRKGNCKKGDACEYAH; CKDGTSCRRRVCFFAH | 12-10-3; 753; 543 |
