# Supplementary material for: Genome-wide analysis of the CCCH zinc finger family identifies tissue specific and stress responsive candidates in chickpea (Cicer arietinum L.)
Source: PLoS One. 2017 Jul 12;12(7):e0180469. doi: 10.1371/journal.pone.0180469 (PMC5507508; doi:10.1371/journal.pone.0180469)
Supplement: S3 Table — (DOCX) [file pone.0180469.s003.docx]

**S3 Table. Details of motifs found in the promoters of CarC3H genes.**

| **Motifs present in promoter** | **Motif sequence** | **Brief description of motif** | ***CarC3H* gene promoters containing motif** |
| --- | --- | --- | --- |
| -300  ELEMENT | TGHAAARK | "TGTAAAG core motif" in "-300 elements" of alpha-zein genes of maize. | All except CarC3H4,24 and 55 |
| 2S SEEDPROT BANAPA | CAAACAC | Conserved in many storage-protein gene promoters; May be important for high activity of the napA promoter | CarC3H2,3,5,6,8,15,18,19,21,23,24,25,26,27,30,40,43,44,46,52,53,54,56 and 58 |
| AACACOREOSGLUB1 | AACAAAC | Core of AACA motifs found in rice (O.s.) glutelin genes, involved in controlling the endosperm-specific expression | All except CarC3H4,5,8,11,23,27,28,33,34,35,36,37,38,44,47,50 and 52 |
| ABREL ATERD1 | ACGTG | ABRE-like sequence required for etiolation-induced expression of erd1(early responsive to dehydration) in Arabidopsis | All except CarC3H4,58,9,11,16,17,19,23,24,27,32,33,36,37,51, 52, and 53 |
| ACGTAT ERD1 | ACGT | ACGT sequence required for etiolation-induced  expression of erd1 (early responsive to dehydration) in Arabidopsis; | All except CarC3H 11,27,32,33 and 53 |
| CARGCW8GAT | CWWWWWWWWG | A variant of CArG motif, with a longer A/T-rich core; Binding site for AGL15(AGAMOUS-like 15); W=A/T | All except CarC3H 23,28,33,40,49 and 50 |
| CBFHV | RYCGAC | Binding site of barley CBF1 and CBF2; CBF= C-repeat (CRT) binding factors; CBFs are also known as dehydration-responsive element (DRE) binding proteins (DREBs);R=A/G; Y=C/T | CarC3H1,3,4,7,8, 10,11,13,14,18,20, 21,22,23,25,26,27, 28,31,32,33,37,38, 39,40,44,45,46,47, 49,51,52,53,56,57 and 58 |
| CCAATBOX1 | CCAAT | "CCAAT box" found in the promoter of heat shock protein genes | All |
| DPBF CORE DCDC3 | ACACNNG | A novel class of bZIP transcription factors, DPBF-1 and 2 (Dc3 promoter-binding factor-1 and 2) binding core sequence; Found in the carrot (D.c.) Dc3 gene promoter; Dc3 expression is normally embryo-specific, and also can be induced by ABA | All except CarC3H18,19,24, 39, 56 and 57 |
| DRE2COREZMRAB17 | ACCGAC | "DRE2" core found in maize (Z.M.) rab17 gene promoter; "DBF1" and "DBF2" bound to "DRE2"; rab17 is expressed during late embryogenesis | CarC3H1,14,21,31,40,44,45,57 and 58 |
| EBOX  BNNAPA | CANNTG | E-box of napA storage-protein gene of Brassica napus | All |
| GAREAT | TAACAAR | GARE (GA-responsive element) | All except CarC3H15,22,23 and 27 |
| LTRE1  HVBLT49 | CCGAAA | "LTRE-1" (low-temperature-responsive element) in barley | CarC3H1,2,3,4,9, 10,11,22,23,24,30, 33,37,44,50,54 and 56 |
| MYB1AT | WAACCA | MYB recognition site found in the promoters of the dehydration-responsive gene rd22 and many other genes in Arabidopsis; W=A/T | All except CarC3H48 |
| MYC CONSENSUSAT | CANNTG | MYC recognition site found in the promoters of the dehydration-responsive gene rd22 and many other genes in Arabidopsis | All |
| NAPIN MOTIFBN | TACACAT | Sequence found in 5' upstream region (-6, -95, -188) of napin (2S albumin) gene in Brassica napus. | CarC3H1,2,3,5,10,11,12,16,21,22,25, 26,27,30,31,32,34, 39,42,45,46,47,48, 49,50,52,54 and 58 |
| NODCON1 GM/  NODCON2  GM | AAAGAT/ CTCTT | Putative nodulin consensus sequences | All |
| OSE1 ROOT NODULE/  OSE2ROOT NODULE | AAAGAT/ CTCTT | Consensus sequence motifs of organ-specific elements  (OSE) characteristic of the promoters activated in infected cells of root nodules. | All |
| PYRIMIDINEBOX | TTTTTTCC/ CCTTTT | "Pyrimidine box" found in the barley (H.v.) EPB-1 (cysteine proteinase) gene promoter; Pyrimidine box found in rice (O.s.) alpha-amylase (RAmy1A) gene; Gibberellin-response cis-element of GARE and pyrimidine box are partially involved in sugar repression | All except CarC3H26,43,47 and 52 |
| RAV1AAT/ RAV1BAT | CAACA/ CACCTG | Binding consensus sequence of Arabidopsis (A.t.) transcription factor, RAV1 | All |
| RYREPEAT ELEMENTS | CATGCA/CATGCAT/CATGCAY | "RY repeat" found in RY/G box of napA gene in Brassica napus (B.n.);Required for seed specific expression."RY repeat motif (CATGCAT)"; Present in the 5' region of the soybean (G.m.) glycinin gene. | All except CarC3H1,3,5,13,21,22,23,24,27,30,39,41,46,49,51 and 56 |
| SEF1MOTIF/ SEF4MOTIFGM7S | ATATTTAWW (W=A/T)/  RTTTTTR (R=A/G) | "SEF1 (soybean embryo factor 1)" binding motif; sequence found in 5'-upstream region (-640; -765) of soybean beta-conglicinin (7S globulin) gene; "SEF4 binding site"; Soybean (G.m.) consensus sequence found in 5'upstream region (-199) of beta-conglycinin (7S globulin) gene. | All |
| TGACGT VMAMY | TGACGT | "TGACGT motif" found in the Vigna mungo (V.m.) alpha-Amylase(Amy) gene promoter; Located between -128 and -123; Required for high level expression of alpha-Amylase in the cotyledons of the germinated seeds. | CarC3H1,2,3,6,8, 12,20,21,22,24,25, 28,30,38,39,43,44 and 56 |
| WRKY71OS | TGAC | "A core of TGAC-containing W-box" of, e.g., Amy32b promoter;  Binding site of rice WRKY71, transcriptional repressor of the gibberellin signaling pathway | All |
| WUSATAg | TTAATGG | Target sequence of WUS in the intron of AGAMOUS gene in Arabidopsis | CarC3H1,2,5,7,8, 12,17,18,21,25,26, 30,31,32,36,41,42, 43,45,46,49,51,54,55 and 58 |
