# Supplementary material for: Genome-wide analysis of the CCCH zinc finger family identifies tissue specific and stress responsive candidates in chickpea (Cicer arietinum L.)
Source: PLoS One. 2017 Jul 12;12(7):e0180469. doi: 10.1371/journal.pone.0180469 (PMC5507508; doi:10.1371/journal.pone.0180469)
Supplement: S4 Table — (DOCX) [file pone.0180469.s004.docx]

**S4 Table. Primer and probe sequences used in this study**

| EF1α | F: TCCACCACTTGGTCGTTTTG  R: CTTAATGACACCGACAGCAACAG |
| --- | --- |
| HSP90 | F: GCAGCATGGCTGGTTACATGT  R: TGATGGGATTCTCAGGGTTGA |
| CarC3H1 | F: CAGGCTGTCAAACCCAGTGTCT  R: CGCGGTCGAGTGGGTGAT |
| CarC3H6 | F: CTCCAAAGACCCCCAATCG  R: GCCGCATGATTGAGATCTGA |
| CarC3H7 | F: GAGAAGGCAGGAGAATGCAGAGA  R: CGCCTCTTGCTGCTCCAATA |
| CarC3H10 | F: CTGCCCAGAACAAGATAATGCA  R: AGATGCCACTGATCTTTTGCAA |
| CarC3H11 | F:GTAACCGCGGCGATACATG  R:GAAGTTGGCGAGGTGTGTG |
| CarC3H14 | F: AAGCTACAGGACTCAAGGGTG  R: CACTGTTGACTCTGCATGTGG |
| CarC3H18 | F: TTATCTGGACAGGGCATTCAATC  R: ACTAGTAGGAATTGCAGGGCTCAT |
| CarC3H19 | F: AGAGTTGGAAAGGAGACGTCTT  R: AGTAAACGGACGAAGGAGATGA |
| CarC3H20 | F: TGCCCCTGATTGTGTTTTGA  R: CCGCGAATAGAAGACACAAAGA |
| CarC3H22 | F: CTGCCAAGATCAGTGTAGAAGC  R: TCTGTTTTGAGTTCACACCACC |
| CarC3H23 | F: GGAAGAAGTAGGCGTTGGAATG  R: GAACCACGGCCAGAATATGAA |
| CarC3H24 | F: GTTACTGTGGGTATGGCGATTC  R: TCAGCTTCATCCCATTCCTTCT |
| CarC3H29 | F: TAGTTCACATTCTTCTGCCCCT  R: GTAAAGTTTTCGCACAGCTTGG |
| CarC3H30 | F: TCTGTTCAGCCCAAAATCGATC  R: TCTTTCTCCTTGGCCTTCTCTT |
| CarC3H31 | F: CCACAACAGACCAACGATTTCC  R: GATCGATGGGTTGACCGTTAGG |
| CarC3H34 | F: TCTTTTCTCAACGAAGGGTCA  R: GAAGAACCTCCCCATCCATAA |
| CarC3H39 | F: CCACCAGTTCCCCCTTATCA  R: TTGTGAAGACCCTGGTTGCA |
| CarC3H40 | F: TCTCAGCCTCTCTAGCTCATATCCT  R: AGCATGCAATCGAAACAGGTATT |
| CarC3H41 | F: CCGACCCGGATTTTGTAGCT  R: GCTCCTCCTCACACCTCTGATC |
| CarC3H45 | F: CAATACTCCACCAAGGAAGCTACTC  R: CGGAGGAATATGGGTCTTCGT |
| CarC3H47 | F: AGGCTTGCTGAGTCATTTGTTACA  R: CTACGTTGGATATTCCTTGGTGATC |
| CarC3H48 | F: TGCTTACGATGAACAAGCGATT  R: GCCATTGGAAGTGAAGCAATTG |
| CarC3H50 | F: CAACTCTTGAAGCAGGGTATGG  R: CAGCTTCATCAGGTCTTTGAGG |
| CarC3H51 | F: GCTTTTAGACACCCTCCATTGG  R: CCGTTGTCTTGTTTGCAGGTACT |
| CarC3H54 | F: AAAAGCCTCTCCCTCCCCTT  R: CCCTAGCAACGTCTCCCCTA |
| CarC3H58 | F: GGATATTGCAAAGTCAGAAGTCGAA  R: CCGGCTGCTTTTGTTGGT |
| CarC3H45_ORF | F: GCC**GGATCC**ATGAGCGGAATACTTTGTGAA  R: ACT**GAGCTC**CATGAGCAAGTCATTGACCCACCC |
| **Probe for Southern** |  |
| CarC3H45_south | F: ACACAACAACAACAACAATAATAATAATAAA  R: TCATTATCACCAGAAGCAGCAT |
| **Yeast one hybrid and transactivation assay primers** |  |
| CarC3H45prom_pAbAi | F: GGC**GAGCTC**AAGAGTCATGGATCGCAACAC  R: AGC**CTCGAG**ATACACCCCGTCAACTCAGG |
| CarABI3_pGADT7 | F: GA**GAATTC**ATGGACTGTGGAGTTGAAT  R: AG**CTCGAG**CATTTAGTATCTTTTCATTTTTTCG |
| CarC3H45ORF_pGBKT7 | F:GC**CATATG**ATGAGCGGAATACTTTGTGAAGAGCAA  R: CG**CCCGGG**TCAAGCCAAGGCAATTTGGTAGCA |
